# Identification and Genome-Wide Prediction of DNA Binding Specificities for the ApiAP2 Family of Regulators from the Malaria Parasite

**DOI:** 10.1371/journal.ppat.1001165

**Published:** 2010-10-28

**Authors:** Tracey L. Campbell, Erandi K. De Silva, Kellen L. Olszewski, Olivier Elemento, Manuel Llinás

**Affiliations:** 1 Department of Molecular Biology & Lewis-Sigler Institute for Integrative Genomics, Princeton University, Princeton, New Jersey, United States of America; 2 Institute for Computational Medicine, Weill Cornell Medical College, New York, New York, United States of America; Seattle Biomedical Research Institute, United States of America

## Abstract

The molecular mechanisms underlying transcriptional regulation in apicomplexan parasites remain poorly understood. Recently, the Apicomplexan AP2 (ApiAP2) family of DNA binding proteins was identified as a major class of transcriptional regulators that are found across all Apicomplexa. To gain insight into the regulatory role of these proteins in the malaria parasite, we have comprehensively surveyed the DNA-binding specificities of all 27 members of the ApiAP2 protein family from *Plasmodium falciparum* revealing unique binding preferences for the majority of these DNA binding proteins. In addition to high affinity primary motif interactions, we also observe interactions with secondary motifs. The ability of a number of ApiAP2 proteins to bind multiple, distinct motifs significantly increases the potential complexity of the transcriptional regulatory networks governed by the ApiAP2 family. Using these newly identified sequence motifs, we infer the *trans*-factors associated with previously reported plasmodial *cis*-elements and provide evidence that ApiAP2 proteins modulate key regulatory decisions at all stages of parasite development. Our results offer a detailed view of ApiAP2 DNA binding specificity and take the first step toward inferring comprehensive gene regulatory networks for *P. falciparum*.

## Author Summary

*Plasmodium falciparum* is the main cause of the devastating human disease malaria. This parasitic organism has a complex lifecycle spanning a variety of different cell types in the mosquito vector and human host. To adapt and survive in these different environments, the parasite precisely regulates the transcription of genes throughout its lifecycle. However, the mechanisms governing transcriptional regulation in *P. falciparum* are poorly understood. To date, a single family of specific transcription factors, the Apicomplexan AP2 (ApiAP2) proteins, has been identified. These DNA binding proteins are likely to play a major role in coordinating the development of this parasite and are therefore of major interest. Here, we determine the DNA binding specificities for the entire *P. falciparum* ApiAP2 family of DNA binding proteins. Our results demonstrate that these proteins bind diverse DNA sequence motifs and co-occur in functionally related sets of genes. By mapping these sequences throughout the parasite genome, we can begin to establish a regulatory network underlying parasite development. This study represents the first characterization of a family of DNA binding proteins in *P. falciparum* and provides an important step towards understanding gene regulation in this parasite.

## Introduction

*Plasmodium falciparum* is responsible for the majority of human malaria cases and causes approximately 1 million deaths every year [1]. The complete lifecycle of *P. falciparum* includes three developmental stages, which occur in its mosquito vector, the human liver, and human blood. Within each developmental stage the parasite undergoes major morphological changes that are accompanied by precisely timed transcription of genes that are necessary for parasite growth, differentiation, and replication. Detailed transcriptome and proteome studies have been conducted across the different stages of the life cycle [2]–[12]. Despite these advances in our understanding of messenger RNA transcript dynamics in *P. falciparum*, very little is known regarding the mechanism of transcriptional regulation, including transcription factor binding and sequence specificity.

Basic transcriptional control in *P. falciparum* appears to resemble that of other eukaryotic organisms, with general transcription factors coordinating the recruitment of RNA polymerase II to core promoter elements [13]–[15]. Experiments aimed at identifying *cis*-acting sequences required for gene expression have successfully identified specific enhancer and repressor sequences upstream of the core promoter elements [16]–[26]. In the asexual blood stage, regulatory sequence elements have been identified for the gene encoding the knob-associated histidine-rich protein (*kahrp*) [16], glycophorin binding protein 130 (*gbp130*) [18], cytidine diphosphate-diacylglycerol synthase (*pfcds*) [19], the DNA polymerase delta gene [20], a subset of the heat shock protein (*hsp*) family [22], the *rif* genes [23] and the falcipains [24]. Additionally, three sequence motifs have been identified upstream of the *var* genes: the SPE1, CPE, and SPE2 motifs, of which the SPE2 motif has been hypothesized to be involved in silencing of *var* gene expression [25], [26]. In sexual blood stage parasites three distinct short sequence elements have been found to regulate expression of the gametocyte genes *pfs16*, *pfs25* [17], and *pgs28* [21]. In addition to these experimentally derived motifs, bioinformatic analyses of the *P. falciparum* genome have identified a number of potential *cis*-elements that may play a role in gene regulation [27]–[36]. However, attempts to identify *trans*-factors have been largely unsuccessful [13], [15], [37], [38], with the exceptions of Myb1 [39], [40] and the high mobility group box (HMGB) proteins [41], [42].

Recently, a large protein family was identified in *P. falciparum*, containing Apetala2 (AP2) domains [43]. AP2 domains were originally described in plants as DNA binding domains approximately 60 amino acids in length [44]. In plants, the AP2 family of transcription factors is one of the largest, playing key roles in developmental regulation [44] and stress responses [45]. The Apicomplexan AP2 (ApiAP2) proteins represent a lineage-specific expansion, and are highly conserved across all *Plasmodium* spp. and in other Apicomplexans including *Theileria*, *Cryptosporidium* [43] and *Toxoplasma* [46]. *P. falciparum* was initially predicted to contain 26 ApiAP2 factors, each containing one to three AP2 domains [43], while in *Toxoplasma* the family is expanded to over 50 ApiAP2 proteins [46]. We have noted a 27^th^ highly conserved ApiAP2 protein (PF13_0267), which agrees with recent Pfam predictions for this protein [47]. Although other DNA binding proteins have been reported in the literature, ApiAP2 proteins represent the largest family of transcriptional regulators identified in *P. falciparum*, where they are expressed throughout the entire developmental lifecycle [43].

Previously, we established that two ApiAP2 proteins, PF14_0633 and PFF0200c, bind DNA with high sequence selectivity [48]. Subsequent work demonstrated that the *P. berghei* orthologue of PF14_0633 (PBANKA_132980) is essential for the formation of sporozoites [49], and specifically regulates sporozoite target genes by binding to the same GCATGCA motif that we identified [48]. More recently, PFF0200c was shown to function as a DNA tethering protein involved in heterochromatin formation and integrity [50] via binding to the previously identified SPE2 motif [25]. Importantly, PFF0200c does not appear to act as a transcriptional regulator in the blood stage. A third study identified a *P. berghei* protein, AP2-O [PBANKA_090590 (PF11_0442)], as an activator of genes required for invasion of the mosquito midgut during the mosquito stage of the life cycle [51]. Together, these studies highlight the importance of the ApiAP2 DNA binding proteins in modulating stage-specific gene regulation and chromatin integrity. Despite these recent advances, the regulatory function of the majority of ApiAP2 proteins remains unknown. The DNA sequences recognized by the members of this protein family are largely uncharacterized, and the target genes that these ApiAP2 factors bind are undefined.

Here we biochemically and computationally characterize the global DNA binding specificities for the entire ApiAP2 protein family from *P. falciparum*. Our results reveal a complex array of DNA sequence elements, with the majority of proteins binding to unique sequences. We demonstrate several cases where multiple AP2 domains within the same ApiAP2 protein are capable of binding distinct DNA sequences. The identification of these unique sequence motifs sheds light on the molecular mechanisms of transcriptional regulation by assigning correlations between putative *cis*-acting sequences in *Plasmodium* and the *trans*-factors that will bind at those sequences genome-wide. Our data reveal the likely identity of *trans*-acting ApiAP2 factors that specifically bind to previously described *cis*-motifs, illuminating some of the previously unknown effectors of plasmodial gene expression. For the many new motifs we identified, we predict putative targets for each of the ApiAP2 proteins. This work represents the first comprehensive analysis of the ApiAP2 DNA binding proteins in *P. falciparum* and provides a crucial missing link toward understanding their role in the regulation of parasite development.

## Results

### *P. falciparum* ApiAP2 Domains Bind Diverse Sequence Elements

The 27 plasmodial ApiAP2 proteins vary drastically in size (Figure S1), however, the predicted 60 amino acid AP2 domains, are well-defined and highly conserved. To determine the DNA binding specificity for the *P. falciparum* AP2 domains, we used protein binding microarrays (PBMs), which enable simultaneous screening of all possible DNA sequences up to ten nucleotides in length without sequence bias [52], [53]. Seminal studies from the Bulyk lab have used PBMs to comprehensively characterize individual transcription factors from a diverse array of organisms including yeast, worm, mouse and human [52]–[54]; and we have previously demonstrated its utility for Apicomplexan AP2 DNA binding proteins [48].

**Figure 1:**
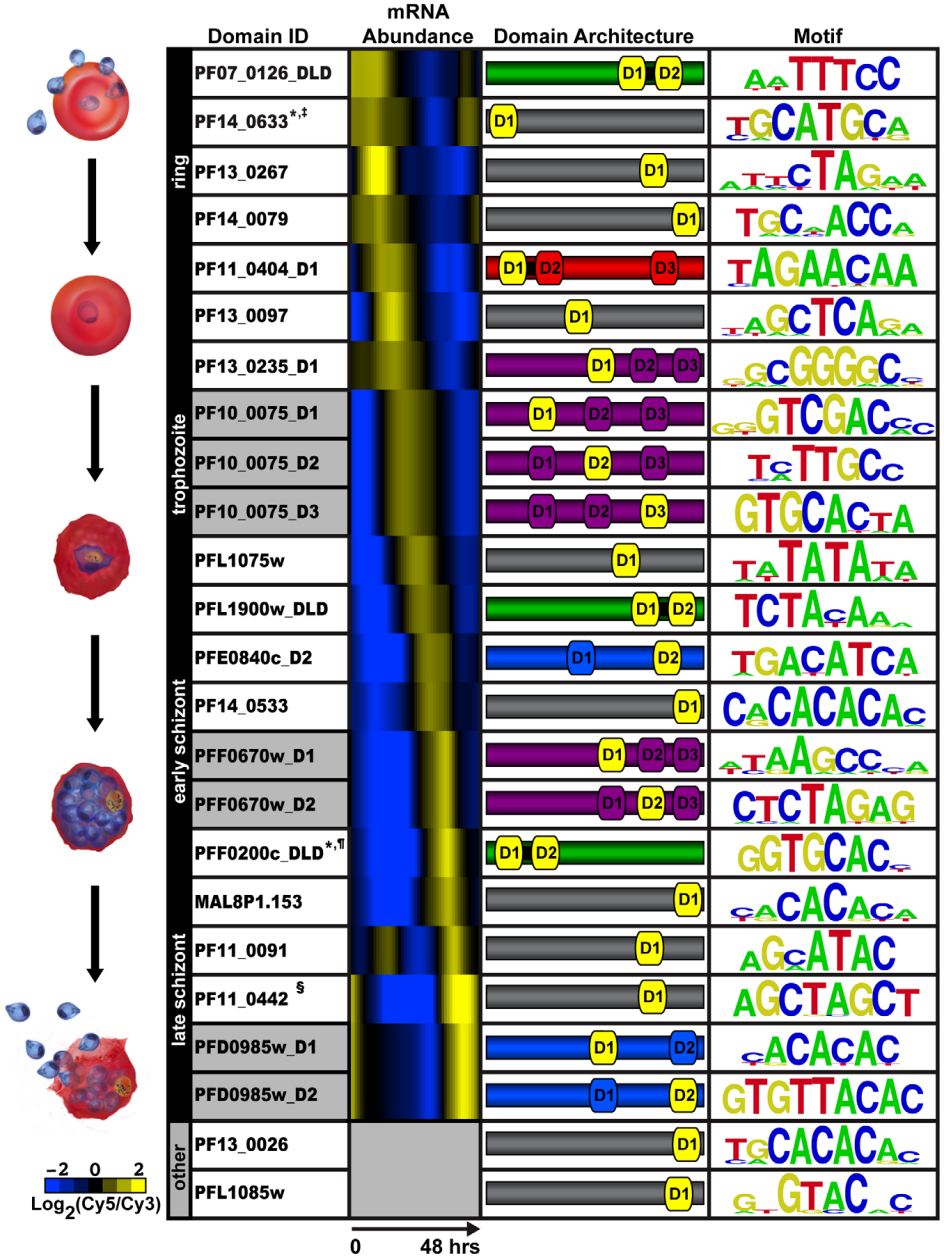
PBM derived motifs for *P. falciparum* ApiAP2 domains. The first column lists the PlasmoDB gene ID and the corresponding AP2 domain tested on the PBM. D1, D2, and D3 refer to the AP2 domains numbered from the N- to C-terminus of each protein; and DLD indicates two domains and a short linker region. Gray shading in this column identifies different AP2 domains from the same protein. Column 2 depicts the relative mRNA abundance profiles for the ApiAP2 genes during the intraerythrocytic developmental cycle (IDC) [2]. Genes that are not expressed during the IDC are in gray. The third column illustrates the number of domains, their architecture and approximate location in the sequence of each ApiAP2 protein (protein sizes are not to scale). The protein colours correspond to the number and arrangement of AP2 domains. Proteins with a single AP2 domain are in gray, two non-tandem AP2 domains are shaded blue, proteins with two tandem AP2 domains are shown in green, triple non-tandem domains are in purple, and the tandem double with an additional third domain is in red. The AP2 domain(s) that binds to the corresponding motif is depicted in yellow. The last column represents the highest scoring motif as a graphical representation of each position weight matrix and visualized using enoLOGOS [92]. Motifs previously identified in the literature are marked as follows: * [48]; ^‡^ [49]; ^¶^ [50]; and ^§^ [51].

We created 50 constructs for PBM screening (Supplemental Text S1, Figure S2), including individual domains, full length proteins, and tandem domain arrangements (two AP2 domains separated by a short conserved linker sequence of 12 to 79 amino acids; designated DLD). Our analysis by PBM of these *P. falciparum* AP2 domains revealed motifs for 20 out of the 27 ApiAP2 proteins (Figure 1), including a motif for the recently identified ApiAP2 protein, PF13_0267, helping to confirm the new annotation for this protein. Results from at least two PBM experiments for each AP2 domain were used to generate position weight matrices (PWMs), which represent the DNA binding affinity for a given domain (Dataset S1, Figure 1). Replicate experiments had excellent correlation coefficients illustrating the robustness of the PBM methodology (see Supplemental Text S1). Enrichment scores (E-scores) were assigned for each 8-mer (allowing up to two gaps) [53], with a significance cut-off of 0.45 (E-scores range from -0.5 to +0.5) for specific 8-mers enriched above background. The E-score is a rank-based, nonparametric score that is robust to differences in protein concentration and reflects the relative preference for each 8-mer [55]. In total we identified sequence motifs for 24 AP2 domains found in a variety of protein architectures (Figure 1). While Figure 1 illustrates which motifs are linked to the blood stage of *Plasmodium* development, several motifs are also associated with ApiAP2 proteins during non-blood stages as well (see Supplemental Text S1). It is noteworthy that different AP2 domains from the same ApiAP2 protein bind distinct DNA sequence elements. However, we do find several motifs that are recognized by multiple ApiAP2 factors (see Supplemental Text S1 and Table S1). This complexity may allow for multifaceted transcriptional regulation using a smaller number of individual factors.

### *P. falciparum* AP2 Domains Can Recognize Multiple Distinct Sequences

Protein-DNA interaction specificities are determined by the chemical interactions of amino acids and DNA bases [56]. Side chain flexibility and DNA distortions allow one DNA binding domain to interact with multiple distinct DNA sequences. For several of the AP2 domains there were significant differences among the top scoring 8-mer sequences that were bound, suggesting multi-motif recognition. Using the Seed and Wobble algorithm [57] we identified alternative motifs associated with 8-mers of high signal intensity that could not be explained by the primary motif for 14 AP2 domains (representing 13 ApiAP2 proteins) (Figure S3, Dataset S2). Some AP2 domains only had a single secondary motif, whereas others had up to four. The secondary motifs can be described based on their relationship with the corresponding primary motifs and fall into the broad categories of end modifications, core changes, variable spacer distances or alternate recognition interfaces [57] (see Supplemental Text S1). The ability of an individual domain to bind anywhere from one to five different DNA sequences would significantly increase the number of target genes that could be regulated by one factor.

**Figure 2:**
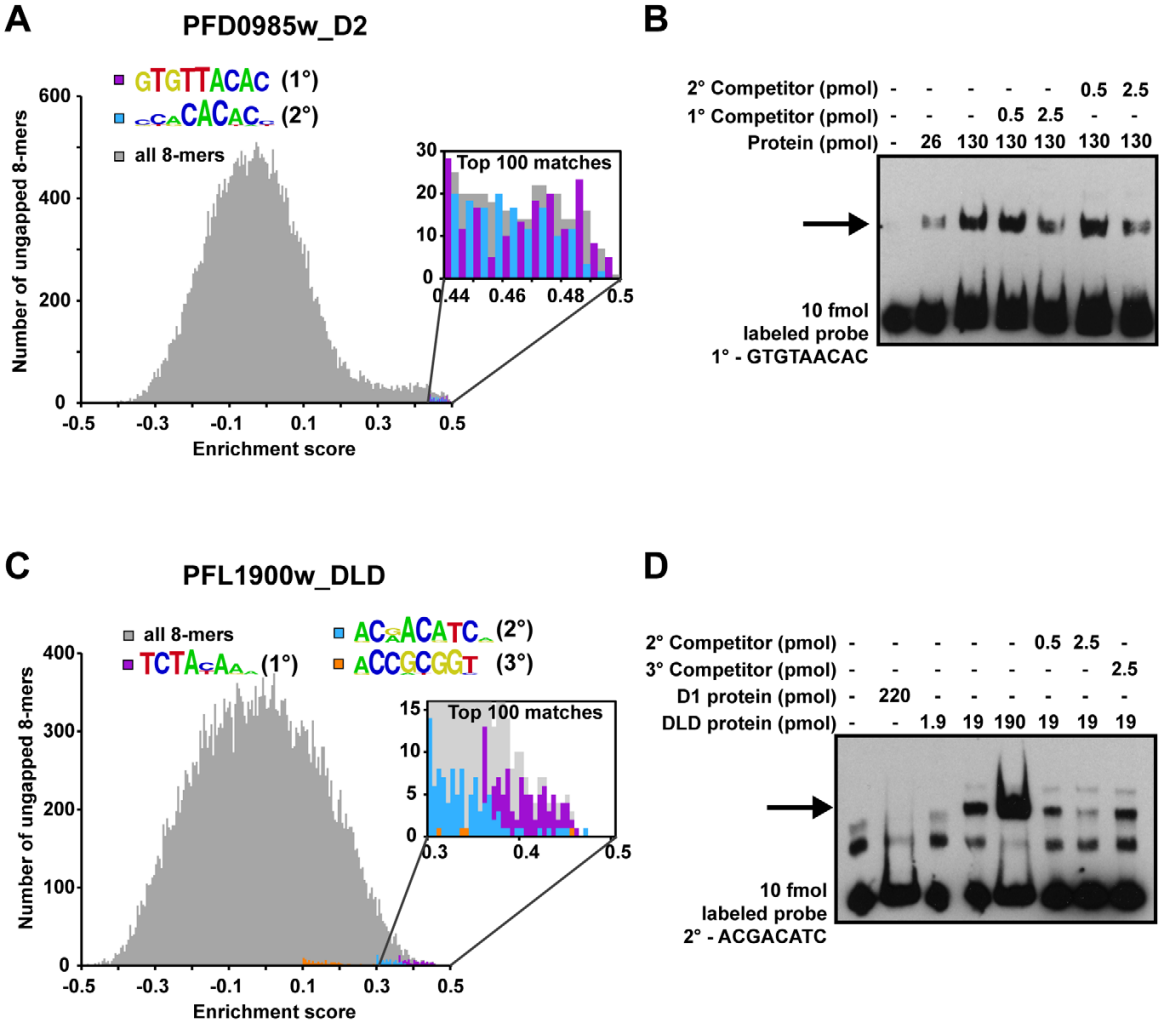
Secondary motifs represent both high- and low- affinity binding sites of ApiAP2 proteins. A) A histogram for PFD0985w_D2 enrichment scores (E-scores) of all ungapped 8-mers (gray) demonstrates that both primary (purple bars) and secondary motifs (blue bars) are enriched in the tail area indicating high affinity interactions. A close-up view (inset) indicates that the top 100 8-mer matches for both motifs show a mixed distribution of E-scores, suggesting that PFD0985w_D2 will bind both motifs equally. B) EMSA competitions between PFD0985w_D2 primary and secondary motifs. The arrow denotes shifted probes. Competition with unlabeled probes of either the primary or secondary motif illustrate that both compete for binding equally well. No binding is observed in the absence of either motif (data not shown). The same experiment using labeled secondary motif probe yielded similar results (data not shown). Probes were labeled with biotin and all competitors are unlabeled. C) A histogram for PFL1900w_DLD E-scores of all ungapped 8-mers portrays similar enrichment of motifs at the high end of E-scores. A close-up view of the top 100 8-mers (inset) reveals that there is a clear preference for the primary motif (purple bars) over the secondary (blue bars), and the secondary motif over the tertiary (orange bars) suggesting different binding affinities. D) EMSA competitions of the PFL1900w_DLD secondary motif with unlabeled probes of the secondary and tertiary motifs, illustrate that the tertiary motif does not compete for binding with the secondary motif. No binding is observed with an unrelated oligonucleotide probe (data not shown). Shifted probes are indicated by the arrow.

We selected two ApiAP2 proteins for confirmation of secondary motif binding by electrophoretic mobility shift assays (EMSAs). Domain 2 of PFD0985w has three predicted secondary motifs in addition to the primary motif. A plot of the E-scores for all ungapped 8-mers reveals that the top 100 matches to both the primary motif and one of the secondary motifs (Figure 2A) are relatively equal in E-score. Therefore, PFD0985w_D2 should bind equally well to these two motifs. To test this hypothesis we generated 60 bp oligonucleotides with the specific motif sequence in the center flanked by random sequences. EMSAs with purified PFD0985w_D2 demonstrate that both oligonucleotides are bound equally well, and that the primary motif is capable of out-competing the secondary motif and vice versa (Figure 2B). No binding is observed with an unrelated non-specific oligonucleotide, indicating specificity for the predicted motifs (data not shown). The second ApiAP2 factor that we selected for confirmation of secondary motifs was PFL1900w_DLD. The highest scoring 8-mers for this tandem domain were represented by completely distinct sequences and a plot of all 8-mers and their E-scores revealed preferential binding with a primary, secondary, and tertiary motif (Figure 2C). PFL1900w_DLD was able to shift all three motifs, but with varying affinities (data not shown for the primary and tertiary motifs), and competition between the secondary and tertiary motifs revealed a clear preference for the secondary motif over the tertiary motif (Figure 2D). These results suggest that the secondary motifs detected represent *bona fide* sequences bound by the AP2 domains and the E-score distributions accurately reflect binding affinities.

### Identification of Putative *Trans*-Factors for Previously Predicted *Plasmodium* Regulatory Motifs

**Figure 3:**
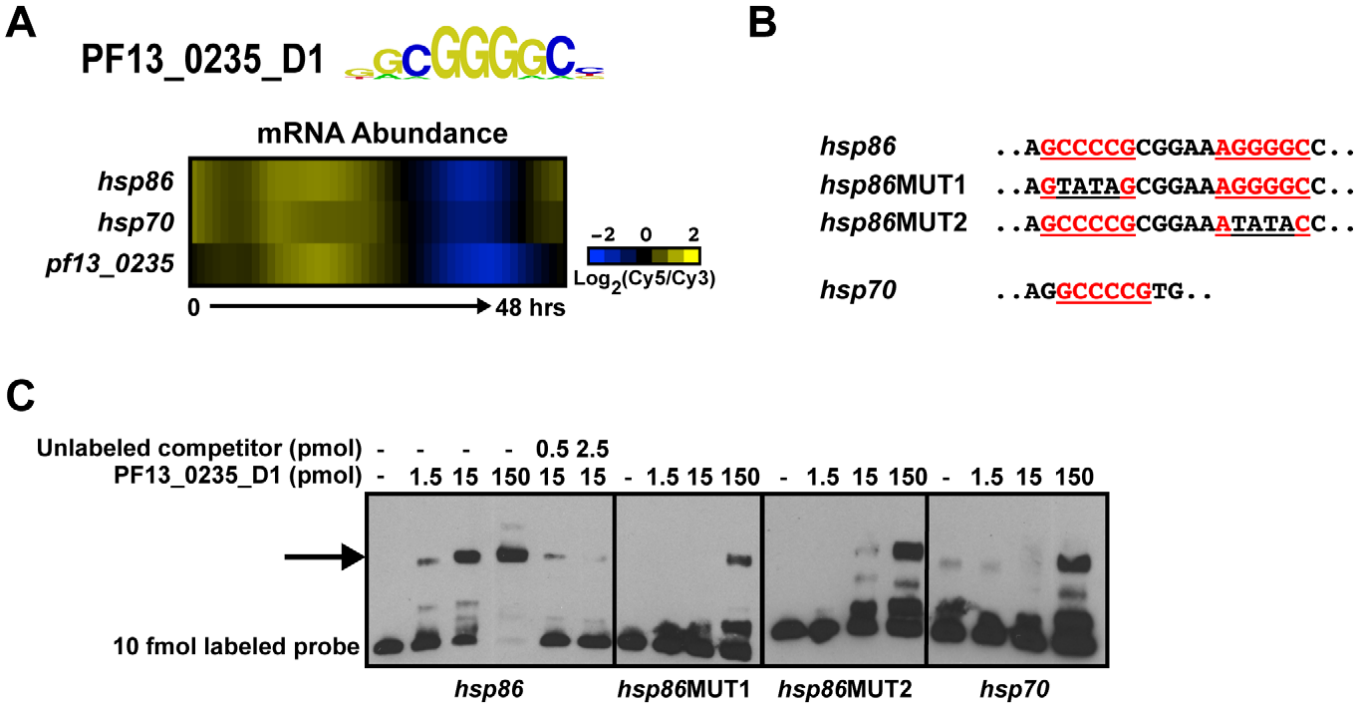
PF13_0235_D1 binds the G-box and is a putative regulator of heat shock genes. A) Transcript abundance profiles of *pf13_0235*, *hsp86* and *hsp70* genes portrays similar timing [2]. B) Partial sequences of EMSA probes from the upstream sequences of *hsp86* and *hsp70*. G-box motifs are underlined and shown in red. Mutations in the G-box sequence are underlined in black. C) EMSAs with probes from the upstream sequence of *hsp86* and *hsp70* illustrate the ability of PF13_0235_D1 to bind the target sequences. Binding to the *hsp86* probe is mediated through both G-boxes as mutation of the first or second sequence significantly reduces binding. No binding is observed with an unrelated oligonucleotide (data not shown). The arrow denotes shifted probes. Probes were labeled with biotin and all competitors are unlabeled.

Both computational predictions and experimental data have identified a number of DNA sequence motifs upstream of genes in *Plasmodium* [17]–[22], [27]–[32], but the specific *trans*-factors that bind to these motifs have mostly remained elusive. For three cases, we now establish plausible links between the newly identified AP2 DNA sequence motifs and these previous reports. Militello *et al*. identified a specific motif, (A/G)NGGGG(C/A) (called the G-box), upstream of 8 out of 18 *Plasmodium* heat shock genes [22]. The occurrence of this GC-rich motif in the genome is low (Table S2), suggesting that its presence in upstream sequences may be significant for transcriptional regulation. The sequence motif that we have identified for PF13_0235_D1 is nearly identical to the G-box element (Figure 3A). Furthermore, the expression profiles of *pf13_0235, hsp86* (*pf07_0029*), and *hsp70* (*pf08_0054*), two heat shock genes containing one or more G-boxes exhibit a strong positive correlation (r = 0.93) during the asexual blood stage [2] (Figure 3A), suggesting that PF13_0235 may play a role in regulating *hsp* gene expression. We performed EMSAs using both G-box elements of the *hsp86* upstream region and found that PF13_0235_D1 interacts specifically with the G-box and deletion of both G-boxes is required to completely eliminate binding (Figure 3B, C). In the presence of only one G-box, binding is severely reduced (Figure 3B, C) suggesting that PF13_0235_D1 preferentially interacts with both G-boxes, perhaps through dimerization (see below). No binding is observed with an unrelated non-specific oligonucleotide at similar protein concentration (data not shown). This result is in agreement with *in vivo* data from transient transfections, where elimination of G-box 1 substantially reduced luciferase expression, but did not completely abolish it [22]. We also tested the G-box from the 5′ flanking region of *hsp70* for *in vitro* binding by EMSA, and confirmed that PF13_0235_D1 binds this sequence *in vitro*. It is interesting to note that the binding of this single G-box motif is similar to that seen for *hsp86* after deletion of one G-box, suggesting that higher affinity interactions require two occurrences of this motif.

Likewise, the sequence element bound by PF10_0075_D3, GTGCA, is enriched in the upstream sequences of genes involved in merozoite development and invasion [31], [58]. Using EMSAs we find that PF10_0075_D3 binds to the GTGCA motif upstream of *msp1* (*pfi1475w*), *msp10* (*pff0995c*) and *rhopH 3* (*pfi0265c*) (Figure S4A), and no binding is observed with an unrelated non-specific oligonucleotide, indicating specificity for the predicted motifs (data not shown). Previous expression studies using a rhoptry gene promoter to drive luciferase expression have demonstrated that the GTGCA motif is important for rhoptry gene-like stage-specific expression [31]. Combined with our EMSA results, this suggests that PF10_0075 may play a role in regulating the expression of invasion-related genes in *P. falciparum*.

Finally, a specific 5 bp motif in the 5′-upstream region of *gbp130* (*pf10_0159*), GTATT, was previously found to be bound by unknown nuclear factors in a sequence-specific manner [18]. The reverse complement of this 5 bp element is nearly identical to the motif we have identified for PF11_0091. EMSAs using the promoter region of *gbp130* and the purified AP2 domain from PF11_0091 confirm its ability to interact with this sequence (Figure S4B), while no binding was observed with an unrelated non-specific oligonucleotide (data not shown), suggesting it is a possible regulator of GBP130 function. The PBM-derived motifs are useful to suggest putative targets for the ApiAP2 *trans*-factors, especially where previous characterization is available. However, *in vivo* assays will be required in all cases to validate these interactions on a protein-by-protein basis.

### Prediction of ApiAP2 Target Genes

To begin to characterize the functional role of ApiAP2 proteins, we searched the *P. falciparum* genome for sequences in promoters and untranslated regions that may serve as regulatory sites for ApiAP2 binding. As a first analysis, we used our AP2-specific position weight matrices generated from the PBM data to search the 5′ upstream sequence elements of *Plasmodium* genes using ScanACE [59], which lists all matches to our position weight matrices within the user defined threshold. Although putative transcription start sites have been predicted [60], actual transcription start sites are still poorly defined in *P. falciparum* [61]. Therefore, we searched 2 kb upstream of the ATG start codon or until an upstream open reading frame was encountered. While this search provides a list of all possible motif occurrences determined from matches to a specific position weight matrix (Datasets S3 and S4), it is undoubtedly an overestimation of putative target genes. In reality, the presence of a regulatory element upstream of a gene does not confirm a regulatory interaction exists, and many motif occurrences may be inactive [62]. Furthermore, for a regulatory element to be functional, it needs to be accessible for binding, which is in part determined by nucleosome occupancy. Nucleosome occupancy has been mapped during the intraerythrocytic developmental cycle (IDC) of *P. falciparum* [63] and using this data we were able to determine that between 65 and 97% of our ScanACE predicted binding sites are accessible (nucleosome-free) at some point during the IDC (Table S2). This suggests that the majority of our predictions have the potential to be active; however, *in vivo* binding affinities may differ from *in vitro* determined affinities, possibly altering the weighting of specific nucleotide positions within the motifs. Ultimately, the actual target sequences of each ApiAP2 protein will need to be individually determined through experimental validation *in vivo* during the specific lifecycle stage of interest.

As a test of the ability of our ApiAP2 proteins to bind to the ScanACE predicted targets we selected a putative target for the newly annotated ApiAP2 protein PF13_0267; *pfc0975c* has a match to the CTAGAA motif at 1469 bp upstream of the start codon. EMSAs showed that the putative target sequence was bound by the purified AP2 domain from PF13_0267, while a mutant oligonucleotide lacking the predicted target sequence did not exhibit significant binding (Figure S5). Although these results demonstrate that our ScanACE-predicted target genes provide a good starting point to search for candidate genes for *in vivo* testing, this does not indicate if *pfc0975c* is a true target of PF13_0267. Indeed the motif bound by PF13_0267 is found upstream of almost all genes and *in vivo* validation will be required to identify actual targets. Complete AP2 motif occurrence data for the *P. falciparum* genome are available to the malaria community at PlasmoDB (www.plasmodb.org) [64].

### Target Gene Refinement Using the IDC Transcriptome

**Figure 4:**
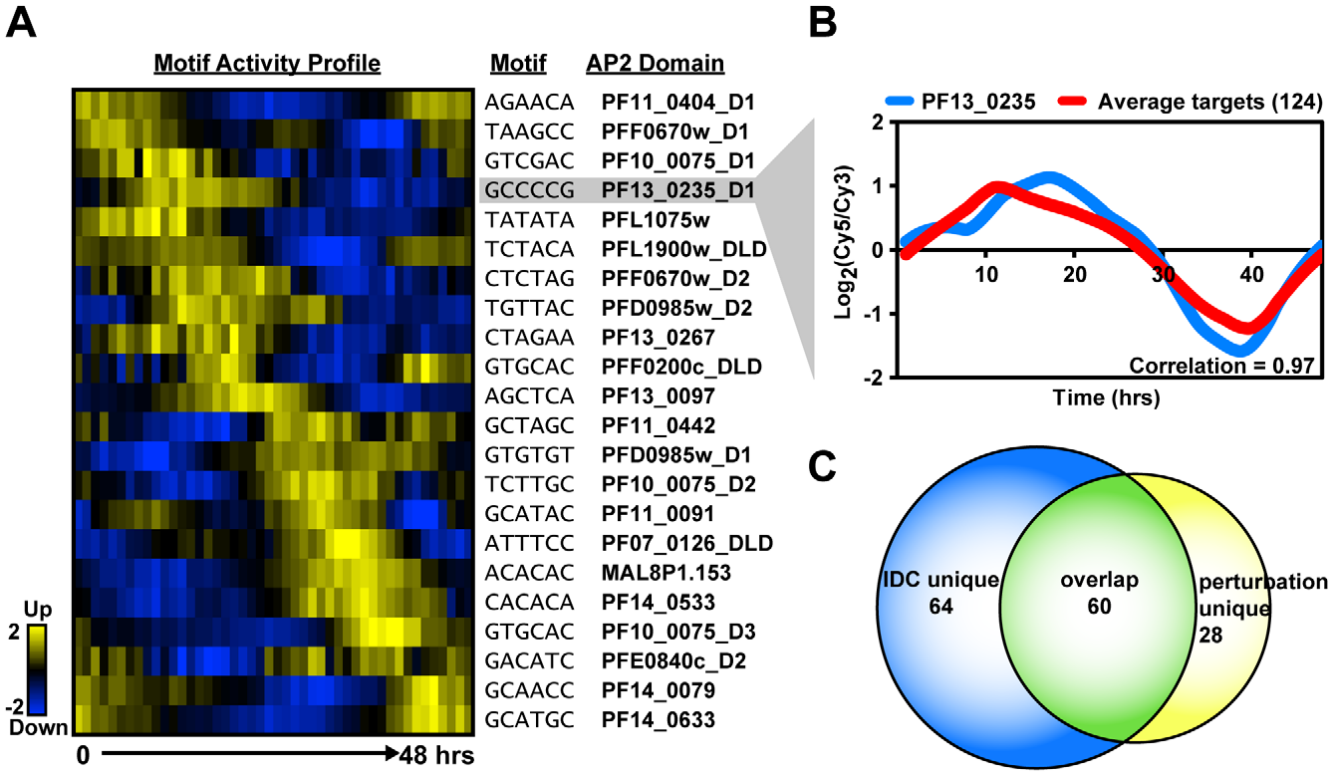
Activity profiles for AP2 motifs and refinement of target gene predictions during the IDC. A) The heat map shows the motif activity profiles based on mutual information content between the motifs and genes expressed at each timepoint of the IDC. Target genes with expression profiles similar to the activity profiles and containing the motif of interest were identified. B) A plot of the average mRNA abundance for target genes with matches to the G-box motif in their upstream sequences is depicted. This is compared with the relative mRNA abundance data for *pf13_0235*, the ApiAP2 factor that binds to the G-box. There is a strong positive correlation (0.97) between *pf13_0235* expression and the expression of its predicted targets. C) The Venn diagram shows the overlap between IDC co-expressed targets and perturbation co-expressed targets. Half of PF13_0235 targets are shared between both datasets, and include ribosomal and heat shock genes.

While the ScanACE analysis provides a list of all occurrences for each motif, it is unlikely that the ApiAP2 proteins are binding to all possible motif occurrences, and instead that they bind to a smaller subset of promoters. Proteins that are co-localized in the cell or form sub-cellular structures such as the ribosome have been found to be transcriptionally co-regulated in other organisms such as yeast, and often are regulated by the same *cis*-elements [65]. Genes that are functionally distinct, but are co-expressed can also be regulated by the same *cis*-elements in their upstream regions. To narrow the ScanACE list to a more informative subset of putative target genes we used relative mRNA abundance profiles to define relationships between co-expressed *Plasmodium* genes [2]. We used linear regression to determine at each time point the extent to which each AP2 motif contributes to (or recapitulates) the overall expression of the genes that contain a given motif in the upstream regions (see Methods and Supplemental Text S1 for details [66]). Thus each motif at each time point is associated with a score (i.e. the fitted regression coefficient), which is positive if genes that have the motif tend to go up at that time point, or negative if they tend to go down. These scores define the predicted motif activity at each time point, and an activity profile across the entire IDC. Activity profiles reflect the predictive effect of individual AP2 motifs on gene expression of a set of target genes at a given IDC timepoint (Figure 4A), and are therefore independent of the mRNA expression profiles of the AP2 genes themselves. The activity profile for each motif was then used to iteratively identify genes containing the target motif in their 5′ upstream regions that share an expression profile similar to the activity profile. This provided a refined list of putative target genes that are co-expressed with one another (Dataset S5).

To illustrate how this approach improved our target predictions we focus on the G-box motif. The ScanACE predicted target list for this motif includes 522 genes (Dataset S3), and using the activity profile-based approach we refine this list to a set of 124 putative co-expressed target genes (Table 1, Dataset S5). A comparison of the average expression profiles for these genes with the expression of *pf13_0235*, the gene for the ApiAP2 factor that binds the G-box, shows a strong positive correlation (0.97; Figure 4). Similar comparisons were made for all ApiAP2 factors and their putative targets (Figure S6), and we observe either significant positive (r-values from 0.97 to 0.43) or negative correlations (r-values from −0.94 to −0.56) for many ApiAP2 factors, implying that this protein family may act as both activators and repressors of target gene expression.

**Table 1:** Number of target genes predicted using the IDC and perturbation refinements, along with the major functional annotations of target genes associated with each motif.

AP2 domain*	Number of targets from IDC or perturbation refinements	Functional annotation of targets [67]	Total number of genes in annotation term	Number of predicted targets in annotation term	P-value	Bonferroni corrected P-value
PF07_0126_DLD	802802	proteasome complex apicoplast	31493	22107	1.3×10^−11^6.0×10^−7^	4.8×10^−9^2.2×10^−4^
PF14_0633	221	protein amino acid phosphorylation	130	14	6.2×10^−6^	0.006
PF13_0267	226226	ribosome biogenesis and assembly purine metabolism	3596	1115	1.3×10^−8^3.5×10^−8^	1.3×10^−5^2.9×10^−6^
PF14_0079	430249	zinc ion binding entry into host	1996	264	9.7×10^−5^4.8×10^−4^	0.080.4
PF11_0404_D1	309531	protein serine/threonine kinase activity zinc ion binding	106199	1631	2.6×10^−5^3.3×10^−5^	0.020.03
PF13_0097	10341034	translation RNA metabolic process	281275	11592	1.5×10^−22^2.5×10^−11^	1.5×10^−19^2.6×10^−8^
PF13_0235_D1	12488	ribosomal subunit response to unfolded protein	9534	144	8.2×10^−8^0.02	3.0×10^−5^1
PF10_0075_D1	24	nucleic acid binding	580	7	0.001	0.7
PF10_0075_D2	949949	DNA replication proteasome complex	6131	3720	1.7×10^−15^1.2×10^−8^	1.8×10^−12^4.3×10^−6^
PF10_0075_D3	283283	entry into host cell nucleosome	612	56	3.2×10^−5^3.5×10^−5^	0.030.01
PFL1075w	10581058	gene expression RNA metabolic process	516275	210116	2.3×10^−50^3.9×10^−26^	2.3×10^−47^3.9×10^−23^
PFL1900w_DLD	617617	RNA metabolic process RNA processing	275119	6133	8.6×10^−14^5.7×10^−10^	8.7×10^−11^5.7×10^−7^
PFE0840c_D2	9898	generation of precursor metabolites and energy mitochondrion	118117	1312	2.5×10^−7^1.7×10^−5^	2.5×10^−4^0.006
PF14_0533	676386	DNA replication post-translational protein modification	61216	2530	3.4×10^−9^3.5×10^−7^	3.4×10^−6^3.5×10^−4^
PFF0670w_D1	772772	gene expression translation	516281	177107	1.1×10^−40^7.9×10^−27^	1.1×10^−38^7.9×10^−24^
PFF0670w_D2	576576	gene expression cytosolic ribosome	51646	13433	4.5×10^−29^4.2×10^−23^	4.6×10^−26^1.5×10^−20^
PFF0200c_DLD	114	gene expression	516	28	1.1×10^−5^	0.01
MAL8P1.153	986986	DNA replication apicoplast	61493	33117	1.9×10^−11^6.2×10^−7^	9.6×10^−9^2.3×10^−4^
PF11_0091	359359	proteasome vesicle mediated transport	3150	1713	8.0×10^−12^8.7×10^−5^	2.9×10^−9^0.08
PF11_0442	223	DNA metabolic process	152	19	1.1×10^−5^	0.01
PFD0985w_D1	710935	DNA replication apicoplast	61493	38132	5.6×10^−22^1.5×10^−12^	5.6×10^−19^5.4×10^−10^
PFD0985w_D2	10241024	gene expression translation	516281	212119	8.0×10^−50^3.9×10^−26^	8.0×10^−47^4.0×10^−23^

*AP2 domains are listed in order of IDC expression as in Figure 1.

Functional annotation of the predicted targets using the DAVID bioinformatics resource [67] identified enrichment of genes involved in specific cellular processes (Table 1). Targets of PF13_0235_D1, the ApiAP2 factor that binds the G-box motif, include genes involved in ribosome function or translation and heat shock response genes. Genes involved in these functions have previously been suggested to be regulated via the G-box element [22], [33], supporting our target gene predictions. Other notable examples include the enrichment of targets involved in cell invasion and host cell entry for the PF10_0075_D3 motif (GTGCAC) and DNA binding for the MAL8P1.153 motif (ACACA). The involvement of the GTGCAC motif in invasion related processes has been independently predicted by three bioinformatic studies [31]–[33], while the ACACA motif was previously associated with DNA replication [31]. Since the majority of these motifs have not been previously described in *P. falciparum*, our prediction of target gene functions are novel and warrant further characterization.

Similarly, we used a recently published *P. falciparum* growth perturbation dataset [68] as an alternative data source to create activity profiles to refine our target gene predictions (Figure S7, Dataset S6, Supplemental Text S1). Genes that respond in a similar manner to a perturbation are more likely to be regulated by the same factor and we observed narrower target gene lists for each motif, many of which overlap with the predictions made using the IDC co-expression data, and others that are novel target gene predictions (Table 1, Figure 4, Figure S8). Further details on the perturbation refinement of target genes can be found in the Supplemental Text S1. Combined, refinement of putative targets using the high resolution temporal gene expression data [2] and the perturbation dataset [68] produce manageable gene sets for further analysis.

### Identification of Motifs Enriched Upstream of *var* Genes

**Figure 5:**
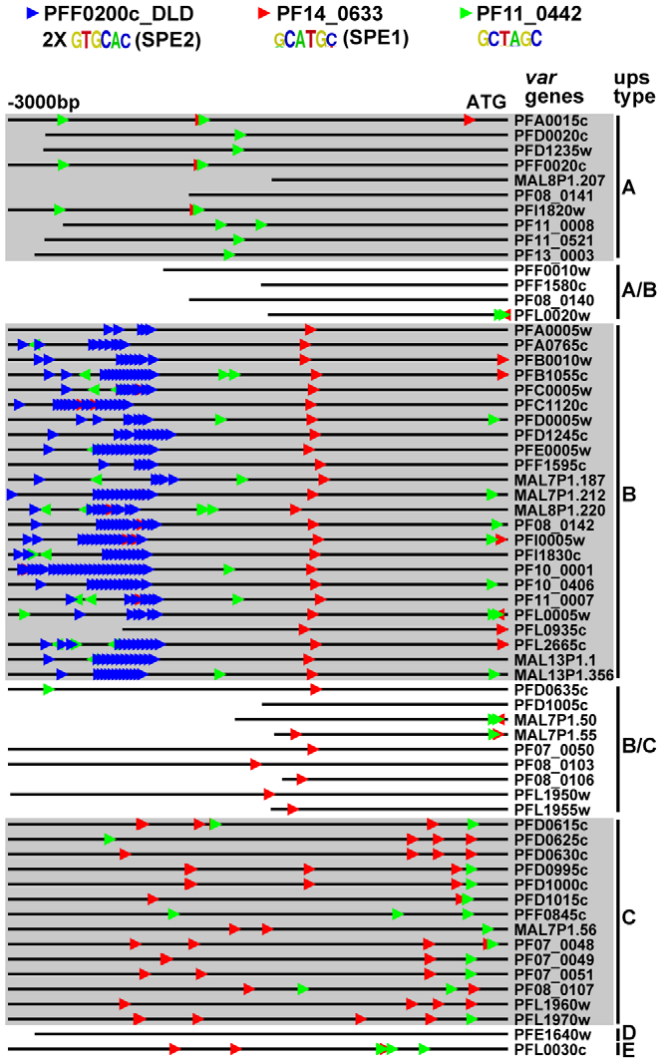
ApiAP2 motifs are conserved in *var* gene promoters. Matches to ApiAP2 motifs were identified using position weight matrices for each motif. Searches were performed on 3 kb upstream of the ATG start codon or until the next upstream gene was encountered (shorter lines). The presence and position of motifs are conserved in the promoters of different types of *var* genes. The previously described SPE1 and SPE2 motifs in upsB *var* genes were found as well as occurrences of the PF11_0442 motif in all types of *var* genes.

We also looked at motif enrichment in the upstream regions of *var* genes. There are approximately 60 *var* genes in *P. falciparum* that encode the antigenically variant erythrocyte membrane protein 1 (PfEMP1), which is involved in sequestration of infected red blood cells in the vasculature [69]. The *var* genes have been divided into groups based on their location along the chromosome (internal or at chromosome ends), the direction of transcription, and the sequences of their intron and 5′ and 3′ untranslated regions [70]. Using our ScanACE prediction of binding sites, we observed a striking pattern of ApiAP2 motifs that were clustered in discrete positions of all three types of *var* promoters (Figure 5). In the upsB promoters we observed repeated motifs for PFF0200c that correspond to the previously identified bipartite SPE2 element [25], located at −2000 to −3000 bp upstream of the ATG start codon. While PF10_0075_D3 binds to a similar sequence as the SPE2 element, recent work has demonstrated that PFF0200c is the primary ApiAP2 factor that binds to this element *in vivo* [50]. We also predict the SPE1 element of upsB *var* genes at −1200 bp [25], which matches to the motif we have identified for PF14_0633. In addition to these previously identified sequence elements, we predict binding sites for PF11_0442, which binds to the sequence GCTAGC (Figure 5). Matches to this motif are conserved in all three major types (A, B and C) of *var* genes. The presence of multiple ApiAP2 binding sites upstream of *var* genes suggests multiple ApiAP2 factors may be recruited for binding upstream of the *var* genes. *var* promoters are silenced by default [71], and it is possible that this silencing is maintained by the co-ordinated action of multiple ApiAP2 factors. Further investigation of these discrete motif-enriched sites will be required to determine the precise role of additional ApiAP2 proteins in *var* gene regulation.

### Target Gene Prediction in *P. vivax*

The IDC transcriptome of *P. vivax* [72] suggests that the regulation of development follows a similar cascade of gene expression as that seen for *P. falciparum* [2], [6]. All *P. falciparum* ApiAP2 proteins have syntenic homologues in *P. vivax* and are expressed at a similar stage of development during the IDC with the exception of *pf14_0471* (*pv118015*) which is shifted from trophozoite to late schizont stage [72] (Figure S9A). AP2 domains are highly conserved across all *Plasmodium spp*. and will likely bind the same motifs. It follows that the ApiAP2 proteins may regulate similar or related target genes in *P. vivax* compared to *P. falciparum*. We calculated activity profiles using the *P. vivax* asexual blood stage transcriptome data [72] (Figure S9B) and as seen for *P. falciparum*, most of the motifs are associated with activation or repression of target genes at one or more timepoints. However, a comparison of the target gene lists for each motif in *P. falciparum* and *P. vivax* shows that the conservation of putative targets is low, ranging from 0 to 53% (Table S3). This implies that although the AP2 DNA binding domains are highly conserved, some of the regulons across these species have diverged and regulation of orthologous genes has evolved independently. It should be noted that a comparison of target genes in non-blood stages, including the mosquito stage, demonstrates substantial statistically significant conservation of motifs among co-regulated genes in *P. vivax* and *P. falciparum* [73]. Therefore it will be important to identify the actual target genes bound *in vivo* for each motif to determine the actual extent of conservation between species.

### Target Genes in Non-Blood Stages of Development

While the above analyses are limited to transcriptional regulation during the blood stage of the lifecycle, ApiAP2 function is not limited to the IDC. Accordingly, we analyzed gene expression data from *P. falciparum* gametocytes, zygotes and sporozoites [6], [10]. Activity profiles for ApiAP2 motifs in gametocyte data revealed activity for a number of the PBM derived motifs during gametocytogenesis (Figure S10A). We found two motifs to be active in zygotes, including the previously identified zygote motif for the *P. berghei* orthologue of PF11_0442 (PBANKA_090590), as well as the CACACA motif, which is bound by PF13_0026 (Figure S10A, Dataset S7). Activity profiles for AP2 DNA motifs in sporozoites identify PF14_0633, PFD0985w_D2, and PFF0670w_D1 as potentially active AP2 domains during this stage (Figure S10A). Since there is currently no liver stage data available for *Plasmodium* species that infect humans, we used data from the rodent malaria species *P. yoelii* [9]. Activity profiles for our PBM derived motifs in the *P. yoelii* liver stage yielded a number of motifs that are potentially active during this stage (Figure S11). Further discussion of the non-blood stage active motifs can be found in the Supplemental Text S1.

## Discussion

Understanding the molecular mechanisms and the regulatory processes that underlie gene expression during the development of *P. falciparum* is a major challenge in malaria research. The ability to map the recognition sites of DNA binding proteins genome-wide enables an improved understanding of how *trans*-factors regulate gene expression and has been undertaken for only a few large eukaryotic families of transcription factors [54], [57], [74]–[76]. Here, we have comprehensively characterized the *P. falciparum* ApiAP2 protein family and established their preferred DNA recognition motifs. A number of findings strongly implicate these factors as major regulators of gene expression in *P. falciparum*. First, the expression of the ApiAP2 genes at distinct times throughout the IDC suggests they are available to regulate target genes throughout the entire 48-hour blood stage of the parasite. Second, the diversity of sequences bound by these domains is sufficient to account for the full cascade of gene expression observed in the IDC. Furthermore, ApiAP2 genes form an interaction network with themselves during the IDC (Figure S12), suggesting that in addition to regulating target genes they may also regulate their own expression. For all three instances where *in vivo* data on binding sites for ApiAP2 proteins are available [49]–[51], our PBM derived motifs are excellent matches, emphasizing the quality of these data and the robustness of the PBMs at identifying preferred sequence elements. Finally, half of the motifs that we identified are nearly identical to motifs that were independently predicted computationally as putative *cis*-elements in the *P. falciparum* genome [27]–[29], [31]–[33].

Our success rate for identifying DNA binding specificities (20 out of 27 ApiAP2 proteins, 74%) using the PBMs is comparable to results obtained using the same technology with other transcription factor families from yeast and mouse (40–85% successful) [54], [57], [76]. For those AP2 domains that did not yield a result, there are numerous technical reasons why we may fail to detect binding. Although we were able to successfully express all of our GST-fusion constructs, possible reasons for failure to detect binding events include insufficient protein concentration, low protein stability, binding conditions (e.g. ionic strength) used, or low-affinity binding (undetectable by PBM). Another possibility is simply that some predicted AP2 domains lack specific DNA binding activity altogether. As mentioned above, the ApiAP2 proteins vary dramatically in size, including four proteins that are less than 40 kDa (Figure S1). Only one of the four smallest ApiAP2 proteins (<300 amino acids) binds to DNA (PF13_0026). Despite our testing both full-length ApiAP2 proteins and isolated AP2 domains from these small proteins, we could detect no DNA binding by PBM. This is surprising given their lack of any other predicted functional domains.

Both computational prediction of motifs [22], [31]–[33] and experimental data [25], [49]–[51] have identified a number of regulatory elements involving repeated iterations of the same motif. One established way to recruit multiple copies of the same factor to a particular site in the genome is through the formation of dimers or multimers of the same protein. A crystal structure has recently been solved of the AP2 domain from PF14_0633, which reveals that the AP2 domain forms a dimer when bound to DNA [77]. This structure suggests that other ApiAP2 proteins may also form homo- or heterodimers [78], thereby recognizing multiple DNA sequence motifs in concert. Further support for this idea is seen in our EMSA analysis of PF13_0235_D1, which shows that higher affinity binding to the G-boxes upstream of *hsp86* (Figure 3) occurs when multiple copies of the motif are present. Similar results were obtained for PF10_0075_D3 and the predicted *rhopH 3* target (Figure S4A) suggesting that AP2 domain dimerization may enhance binding to these sequences. Our ScanACE predictions of target genes identify motif repeats in upstream regions (Dataset S3), which may allow for tighter control of target gene expression by the ApiAP2 factors. Similarly, heterodimer formation may facilitate combinatorial regulation of gene expression at co- motifs. Evidence for such interactions can be found in yeast two-hybrid assays that have detected associations between ApiAP2 proteins [79]. Taken together, our genome-wide motif predictions and the ability of plasmodial AP2 domains to form dimers implies that these factors likely work together to regulate target gene expression.

The extremely high level of conservation (>95% identity) for each AP2 domain across all *Plasmodium spp.* [43] suggests that orthologues from other species will bind to similar DNA sequence motifs. Indeed, motifs for the *P. falciparum* AP2 domains of PF14_0633 and PF11_0442 are matches to the experimentally determined motifs for their *P. berghei* orthologues (AP2-Sp and AP2-O, respectively) [49], [51]. However, while the *cis*-elements bound by individual AP2 domains may be conserved across species, our predicted IDC target gene sets for ApiAP2 proteins appear to differ extensively. This is common in other eukaryotic organisms, where DNA binding domains are highly conserved across species, but downstream target genes are divergent [80]–[85]. Our data suggests that this may be true for *Plasmodium spp*., as demonstrated by the divergence of IDC target gene sets in *P. vivax* and *P. yoelii* compared to *P. falciparum*, which contrasts with the almost perfect conservation of AP2 domains and the similar temporal expression of ApiAP2 genes. We previously showed that the orthologous AP2 domains from PF14_0633 in *P. falciparum* and its distant Apicomplexan relative *C. parvum* (cgd2_3490) bind virtually identical sequence elements [48], but their predicted regulons had virtually no overlap. However, there are some examples of transcription factor binding site conservation among specific groups of target genes. For example, the G-box element has been predicted to regulate heat shock genes in both *C. parvum* [86] and *P. falciparum* [22], and both the PF14_0633 (TGCATGCA) and PF10_0075_D3/PFF0200c_DLD (GTGCAC) motifs are conserved among sporozoite and merozoite invasion genes in *P. falciparum*, *P. vivax*, *P. yoelii*, and *P. knowlesi* [87]. A better assessment of target gene conservation between species will be possible with more accurate target gene lists from chromatin immunoprecipitation experiments for each individual ApiAP2 factor. Indeed, this has recently been demonstrated in the asexual blood stages for PFF0200c [50], and only a subset of predicted targets were actually bound *in vivo* by this factor. This is also likely to be true for other ApiAP2 factors and further work identifying functional binding sites will clarify the level of conservation of transcription factor binding sites among the different *Plasmodium spp*.

Combinatorial gene regulation is an important aspect of transcription in many organisms. It controls the level of gene expression, the precise timing of expression, and determines the ability of a regulatory circuit to respond differently to a wide variety of extracellular signals. The finding that there are a relatively small number of specific transcription factors in *P. falciparum* prompted the hypothesis that combinatorial gene regulation plays an important role in the parasite [29]. Our finding that some ApiAP2 proteins can bind more than one sequence element significantly increases the potential complexity of the regulatory network. We identified secondary DNA binding preferences for 14 AP2 domains. These secondary motifs allow one AP2 domain to regulate a much broader range of targets than initially predicted from our ScanACE analysis using only the primary motifs. Precedent for this has been seen in yeast where the transcriptional activator, HAP1, binds two completely different regulatory sequences, allowing for the regulation of target genes with different promoter elements [88]. A similar observation was made in a recent PBM analysis of 104 mouse transcription factors, where it was found that almost half of the TFs recognized multiple sequence motifs [57]. A re-analysis of previously generated chromatin immunoprecipitation – microarray (ChIP-chip) data illustrated that these newly identified alternate motifs were also bound *in vivo* [57]. Similar *in vivo* data will be required to fully elucidate the role of individual ApiAP2 motif occurrences on target gene functions; however it is evident from our data that the ApiAP2 factors have sufficient diversity in sequence recognition to potentially regulate all *P. falciparum* genes.

While it is clear that the ApiAP2 factors bind DNA and recent work has begun to explore their contribution to transcriptional regulation [49], [51], these factors are also capable of binding DNA as scaffolding and recruitment proteins [50]. This finding opens up new possible functions for this family of DNA binding proteins. The next step in understanding ApiAP2 function will be to address the role of other domains in these proteins. The majority of ApiAP2 proteins are predicted to encode extremely large proteins, yet nothing is known regarding other functional domains outside of the AP2 domain. Yeast two-hybrid assays have identified interactions between ApiAP2 proteins and chromatin modifying proteins [79], which could help recruit ApiAP2 proteins to target sites in the genome. How and when ApiAP2 proteins are targeted to the nucleus and if this is actively regulated also remains unanswered. Our global mapping of ApiAP2 motif preferences represents the first characterization of a *Plasmodium* family of DNA binding proteins and provides a starting point to investigate transcriptional control in *P. falciparum*. These results provide an important step toward understanding the role of these proteins as major regulators throughout all stages of parasite development in *Plasmodium spp*. and other related Apicomplexan species.

## Materials and Methods

### Cloning, Expression, and Purification of *P. falciparum* ApiAP2 Domains

Domain boundaries were defined as in [43] and extensions were made based on sequence homology both 5′ and 3′ of the AP2 domains amongst *Plasmodium spp.* orthologues, as well as using structural predictions from the online secondary structure prediction server Jpred3 [89]. In total 77 different versions of ApiAP2 domains from *P. falciparum* as well as one from *P. berghei* were cloned into pGEX-4T1 (GE Life Sciences) to create N-terminal glutathione S-transferase (GST) fusions (Figure S2). Proteins were expressed in BL21-CodonPlus(DE3)-RIL cells (Stratagene) with 0.2 mM IPTG at room temperature and affinity purified using glutathione resin (Clontech). The purity of each protein was estimated by silver stained SDS-PAGE and yields were calculated based on absorbance at 260 nm and specific molar extinction coefficients. All protein concentrations include contaminating products and will be an overestimation of the actual AP2 domain amounts.

### Protein-Binding Microarrays (PBMs)

All constructs in Figure S2 were tested at least once on the PBMs, and positive motifs were confirmed at least twice on independent arrays. The PBM experiments were performed as previously described [53]. Briefly, custom designed oligonucleotide arrays are double-stranded using a universal primer, incubated with GST-AP2 fusion proteins, visualized with Alexa-488 conjugated anti-GST antibody, and scanned using an Axon 4200A scanner. Proteins were used at the maximum concentration obtained from purification and represent one-fifth of the total reaction volume used on the PBM. In this study three different universal platforms were used covering all contiguous 8-mers as well as gapped 8-mers spanning up to 10 positions. After data normalization and calculation of enrichment scores [53], [55] the “Seed-and-Wobble” algorithm was applied to combine the data from two separate experiments and create position weight matrices (PWMs) [55]. An enrichment score cut-off of 0.45 was used to distinguish high affinity binding data from low affinity and non-specific binding. The score for each 8-mer reflects the affinity of a DNA binding domain for that sequence, with higher scores representing tighter interactions [55]. Secondary motifs were identified by running the “rerank” program until E-scores below 0.45 were obtained [55]. The PBM analysis suite was downloaded from the Bulyk lab (http://the_brain.bwh.harvard.edu/PBMAnalysisSuite/index.html). For public access, all motifs have been deposited in the UniPROBE database [90].

### Electrophoretic Mobility Shift Assays (EMSAs)

N-terminal GST fusions of the ApiAP2 domains were purified as described above. Single-stranded HPLC purified 5′ biotinylated oligonucleotides were purchased from Integrated DNA Technologies (http://www.idtdna.com) and annealed with complementary oligonucleotides to create double-stranded probes (Table S4). EMSAs were performed using the LightShift Chemiluminescent EMSA kit (Pierce). Briefly, purified protein was incubated with 50 ng/µL of poly(dI-dC) and 10 fmol of biotinylated probes. Competitor DNA was added in 50 or 250 fold molar excess. All reactions were incubated in the kit EMSA buffer with 2.5% glycerol, 5 mM MgCl_2_, 10 mM EDTA, 50 mM KCl, and 0.05% NP-40 at room temperature for 20 minutes. Electrophoresis and transfer to Nylon membrane (Hybond) was performed according to the manufacturer's instructions. The Chemiluminescent Nucleic Acid Detection Module (Pierce) was used according to the manufacturer's instructions to visualize the probes.

### Identification of Potential Target Genes Using PBM Derived Position PWMs

2 kb-long upstream regions (or up to the nearest ORF) were first extracted from whole genome sequences and associated gene annotation data (PlasmoDB 6.0). PBM motifs were trimmed down to their 6 most informative consecutive motif positions (motif cores). Then, we determined all core PBM motif occurrences in the 2 kb regions using the ScanACE approach [91]. G+C content was set to 13.1% in *P. falciparum*, 42.8% in *P. vivax* and 20.1% in *P. yoelii*. Score threshold was set to the average score of randomly drawn sequences from the PBM PWMs, minus two standard deviations (this is the default ScanACE setting).

### Refinement of Target Gene Lists

In order to identify candidate target genes for each AP2, we reasoned that these target genes should be co-expressed and should share the AP2 binding site we identified using PBMs. Thus, we first identified groups of genes that are co-expressed across multiple experimental conditions or multiple time points (e.g., in the IDC). Then for each AP2 motif, we determined in which of the groups the motif was over-represented. For each of these groups, we extracted the genes associated with one or more motif occurrences. Thus, target genes in this definition can come from multiple co-expression groups (and not all genes in these co-expression groups end up in the target list because not all genes in these groups will have a motif occurrence in their promoter). In order to define co-expressed gene groups, we used the *k*-means approach together with the Pearson correlation. Motif scanning was performed using the ScanACE approach [91] as described above. In order to determine functional motif score threshold, we used an information-theoretic procedure analogous to that used in FIRE [27]: briefly, we determined the motif score threshold such that the resulting motif occurrences best explain the co-expression clusters obtained by *k*-means. At a given motif score threshold, motif over-representation in each cluster was assessed using the hypergeometric distribution; to correct for multiple testing (i.e. multiple clusters being evaluated), we used the Benjamini-Hochberg procedure; corrected p-values corresponding to an estimated overall FDR of 0.25 were considered significant and the genes associated with motif occurrences in these clusters were extracted. Because the *k*-means is dependent on initialization, we repeated the entire procedure 10 times; genes extracted 3 times or more (out of 10) were considered as candidate target genes. Thus, only genes associated with the considered motif and that are consistently found as co-expressed together with other genes sharing that same motif end up as candidate target genes in our analysis. More detailed methods are available in the Supplemental Text S1.

### Accession Numbers

PlasmoDB (www.plasmodb.org) accession numbers for genes and proteins discussed in this publication are: *hsp86* (PF07_0029); *hsp70* (PF08_0054); *msp1* (PFI1475w); *msp10* (PFF0995c); *rhopH 3* (PFI0265c); *gbp130* (PF10_0159); AP2-O (PBANKA_090590); AP2-Sp (PBANKA_132980).

## Supporting Information

Supplemental Text S1 Supplemental results and discussion.
(0.13 MB PDF)

Figure S1 Size distribution of ApiAP2 proteins. Proteins range in size from 200 amino acids to over 4000, with the four smallest ApiAP2 proteins having less than 500 amino acids. Bars are colour coded based on the number of AP2 domains in each protein.
(0.38 MB TIF)

Figure S2 ApiAP2 domains tested on PBMs. ApiAP2 proteins are listed in order of size. Domains were cloned into pGEX-4T1 to produce N-terminal GST fusions. Proteins were expressed and purified from *E. coli* and tested in duplicate on protein binding microarrays (PBMs). D1 indicates the AP2 domain closest to the N-terminus; D2 and D3 are the AP2 domains following D1 going from the N- to C-terminus of the protein; DLD indicates two domains and a short linker region (Domain - Linker - Domain); ext at the end of the domain number indicates an extension of the original cloned domain at either or both of the N- and C-termini. Enrichment scores above 0.450 were considered significant, and no result indicates an E-score below this cut-off. PFL1900w_DLD has a poly-asparagine tract that increases its linker length by 39 amino acids. This expanded linker is absent in the *P. berghei* orthologue (PB000218.00.0) of PFL1900w, while the AP2 domain sequences are 99% identical. To test the effect of the PFL1900w expanded linker on DNA binding we generated a GST fusion DLD construct for the shorter *P. berghei* orthologue. Both constructs exhibited identical DNA binding specificity.
(0.26 MB PDF)

Figure S3 AP2 domain secondary motifs. Secondary motifs found for ApiAP2 proteins and their associated enrichment scores. E-scores greater than 0.450 were considered significant. The final column lists the relationship of the secondary motif to the primary motif using the following descriptions: end modification is a change in nucleotide specificity at either or both the 5′ and 3′ ends of the motif, alternate recognition interface is a motif that is unrelated to the primary motif, variable spacer distance is an insertion or deletion in the middle of the motif, and core change is a change in nucleotide specificity in the middle of the motif.
(0.26 MB PDF)

Figure S4 PF10_0075_D3 and PF11_0091 bind *Plasmodium* regulatory motifs. A) Partial sequences of the EMSA probes from the upstream sequences of *rhopH3*, *msp1* and *msp10*. The PF10_0075_D3 motif is underlined in red and mutations are underlined in black. EMSAs using these probes illustrate the ability of PF10_0075_D3 to specifically bind the target sequences (shifted complexes are denoted with an arrow). No binding was observed with an unrelated oligonucleotide. Probes are biotin labeled and all competitors are unlabeled. B) EMSA using the *gbp130* upstream sequence demonstrates that PF11_0091 binds to this sequence, but not to a non-specific probe. Sequences are indicated as in (A).
(1.84 MB TIF)

Figure S5 PF13_0267 binds to a sequence upstream of a ScanACE predicted target gene. A) Transcript abundance profiles of *pf13_0267* and a predicted target gene, *pfc0975c*, show similar timing [7]. B) EMSA using the upstream sequence of *pfc0975c*, a putative target of PF13_0267. Biotinylated probe is specifically shifted with increasing amounts of the purified protein (designated by the arrow) and this shift is competed with unlabeled probe DNA. No shift is observed with an unrelated oligonucleotide (data not shown). Partial probe sequences are shown below the gel, with the PF13_0267 target motif underlined in red and mutations of the motif underlined in black.
(0.63 MB TIF)

Figure S6 Correlation of ApiAP2 mRNA abundance and expression of putative target genes. Average targets represents the average mRNA abundance profiles during the IDC for all genes in Dataset S5. mRNA abundance profile data was taken from [7]. The correlation coefficients are provided in the bottom right of each plot.
(1.01 MB PDF)

Figure S7 Activity profiles for AP2 motifs using perturbation data. To refine our list of target genes we defined activity profiles for each motif using the IDC perturbation data [16]. Activity profiles are grouped by drug treatments and their corresponding controls. Each row represents the motif activity profiles and timepoints are from left to right within each treatment. Specific details for each perturbation experiment can be found in [16].
(2.25 MB TIF)

Figure S8 Overlap between predicted IDC and perturbation co-expressed targets. Blue circles represent the predicted IDC co-expressed targets and the yellow shaded circles are the perturbation co-expressed targets. The overlap between the two gene lists is shown in green. The numbers indicate the number of unique gene IDs in each dataset. The AP2 domain that binds to the corresponding motif is listed above each Venn diagram.
(4.62 MB TIF)

Figure S9 ApiAP2 IDC expression and activity profiles for the AP2 motifs in *P. vivax* samples. A) A comparison of IDC mRNA abundance profiles for the *P. falciparum* ApiAP2 proteins [7] with motif data and their *P. vivax* orthologs [29]. Expression is similar between the two species. Gray indicates data not available. B) To compare target genes for each motif in *P. falciparum* and *P. vivax*, activity profiles were defined using three *P. vivax* isolates [29]. The columns in the heat map represent the nine timepoints and rows are the motif activity profiles. ApiAP2 proteins are ordered as in Figure 4.
(1.85 MB TIF)

Figure S10 Activity profiles for AP2 motifs in different stages of the *P. falciparum* lifecycle and target gene predictions. To identify motifs that function in different stages we used data from across the lifecycle [8], [10]. Motif activity profiles for the IDC are in duplicate, using either sorbitol or temperature synchronized parasites. Data for gametocyte expression is from a 14 day experiment and zygotes, ookinetes, and sporozoites represent a single timepoint. Each row is the activity profile for an AP2 motif.
(1.04 MB TIF)

Figure S11 AP2 motif activity profiles during the *P. yoelii* liver stage. Motifs for the *P. falciparum* ApiAP2 proteins were used to establish activity profiles in the *P. yoelii* liver stage (LS) [20]. Columns are grouped based on the hour post invasion in the liver (24, 40, or 50 hours). At each timepoint LS samples were compared to a range of samples: mosquito oocyst sporozoites (ooSpz), mosquito salivary gland sporozoites (sgSpz), to alternate LS timepoints, and to blood stage schizonts (sSchz) and mixed blood stage samples (BS). ApiAP2 proteins are ordered as in Figure 4.
(1.26 MB TIF)

Figure S12 *P. falciparum* IDC ApiAP2 regulatory network. ApiAP2 genes are targets of other ApiAP2 factors. ApiAP2 genes are arranged in order of expression during the IDC in a clockwise manner starting at PF07_0126. Arrows pointing away from an ApiAP2 gene indicate that it potentially regulates the target factor by binding to motifs in the target upstream region. ApiAP2 genes coloured in green are expressed during the IDC, but did not exhibit DNA binding specificity on the PBMs. Genes in yellow are expressed during the IDC and bind to specific DNA sequences and genes in blue also bind DNA, but are not expressed during the IDC.
(0.67 MB TIF)

Figure S13 ApiAP2 proteins that bind the CACACA motif. A) Three of the ApiAP2 factors that bind the CACACA motif are expressed in the late stages of the IDC as shown by mRNA abundance profiles [7]. B) An alignment (performed using ClustalW; www.ebi.ac.uk/clustalw) of the AP2 domains for these three factors demonstrates a high level of similarity (52%) in the predicted β-sheets (gray arrows above the alignment); which likely contain the DNA binding residues. Identical residues are highlighted in red and similar residues in yellow. Secondary structure predictions were made using Jpred3 [5]. Addition of PF13_0026 to the alignment, the one CACACA-binding factor that is not expressed in the IDC, shows that the sequence of this AP2 domain is more divergent. (C) Phylogenetic tree of the predicted β-sheet regions of the AP2 domains. The tree demonstrates that the three IDC expressed ApiAP2 factors that bind the CACACA motif are more similar to one another than to any other AP2 domain. The tree was made using PhyML, using maximum likelihood with a LG protein evolution model.
(0.84 MB TIF)

Table S1 Similarity E-values calculated using the STAMP tool between ApiAP2 position weight matrices.
(0.02 MB XLS)

Table S2 Distribution and accessibility of ApiAP2 binding sites.
(0.05 MB PDF)

Table S3 Conservation of IDC ApiAP2 targets between *P. falciparum* and *P. vivax*.
(0.06 MB PDF)

Table S4 Oligonucleotides used in EMSAs.
(0.08 MB PDF)

Dataset S1 Position weight matrices for all AP2 domains that bound DNA in PBM experiments.
(0.06 MB XLS)

Dataset S2 Position weight matrices for all secondary AP2 motifs.
(0.10 MB XLS)

Dataset S3 ScanACE results for all AP2 motifs. ScanACE results for all ApiAP2 motifs. The location, orientation, and sequence of each motif match in upstream regions are indicated.
(8.80 MB ZIP)

Dataset S4 Motif occurrence across the *P. falciparum* genome.
(2.12 MB XLS)

Dataset S5 IDC co-expressed predicted targets for all motifs.
(1.71 MB ZIP)

Dataset S6 Perturbation co-expressed predicted targets for all motifs.
(1.49 MB ZIP)

Dataset S7 Sporozoite and zygote predicted targets of ApiAP2 proteins.
(0.18 MB ZIP)
