# Supplementary material for: Identification and Genome-Wide Prediction of DNA Binding Specificities for the ApiAP2 Family of Regulators from the Malaria Parasite
Source: PLoS Pathog. 2010 Oct 28;6(10):e1001165. doi: 10.1371/journal.ppat.1001165 (PMC2965767; doi:10.1371/journal.ppat.1001165)
Supplement: Supplemental Text S1 — Supplemental results and discussion. (0.13 MB PDF) [file ppat.1001165.s001.pdf]

Supplemental Text

Results and Discussion

Cloning AP2 domains for protein binding microarrays (PBMs)

PBM experiments require the protein of interest to be cloned with an epitope tag, purified, and then incubated with a commercial double-stranded DNA microarray [1]. We therefore created 50 N-terminal GST fusion constructs for the AP2 domains in *P. falciparum* (Figure S2) and measured their DNA binding affinities using PBMs [1-3]. These 50 constructs include 42 individual domains cloned as small, 60 amino acid proteins, four full-length proteins, and four constructs that we have designated as domain-linker-domain (DLD) (Figure S2). In *P. falciparum* there are five ApiAP2 proteins that have two AP2 domains located adjacent to one another (tandem double domains) and are separated by a highly conserved linker sequence (ranging in size from 12 to 79 amino acids). A similar arrangement is seen for plant AP2s, where both domains in a tandem arrangement are required for DNA binding [4]. To test if both domains are required in *P. falciparum* ApiAP2 proteins, we cloned the DLD arrangement for four of the five tandem double domains (PF07_0126, PFF0200c, PFL1900w, and PF11_0404) and cloned the full-length protein for the fifth (PF11_0163). After initial screening of the 50 purified proteins, a large fraction did not bind well on the PBM. We subsequently re-optimized our gene expression constructs for 23 AP2 domains, to include flanking residues that are likely to be important for domain stabilization as predicted by Jpred [5] and re-tested these new constructs (Figure S2).

PBM Reproducibility

To analyze the reproducibility of the PBMs we compared the position weight matrices (PWMs) from replicate experiments using the approach previously described in CompareACE [6]. This method calculates the Pearson correlation between the weight of the weight matrices. Two motifs with a CompareACE score greater than 0.7 are considered similar, while a score of 1.0 indicates the motifs are identical. Using data from four replicates performed on different days, the PWMs for PF13_0026 give Pearson correlation values all greater than 0.9 indicating the motifs are nearly identical and the PBM data is highly reproducible.

Motifs associated with the blood stages of development

The majority of the binding motifs that we have identified are associated with ApiAP2 proteins that are transcribed at specific stages of the intraerythrocytic development cycle (IDC) in *P. falciparum* (Figure 1). Each of the major stages in the IDC (ring, trophozoite, early schizont, and late schizont) is represented and contains at least three different ApiAP2 proteins with specific binding preferences. ApiAP2 relative transcript abundance profiles recapitulate the full cascade of gene expression previously described for the IDC [7,8] and implies that ApiAP2 proteins function in each stage of the IDC to regulate transcription. Within each stage there are slight differences in the temporal expression patterns for each ApiAP2 gene, suggesting that each individual factor controls a distinct subset of genes at a given stage of development.

Motifs associated with ApiAP2 proteins in non-blood stages of development

While the majority of the *P. falciparum* ApiAP2 proteins are expressed during the IDC, several ApiAP2 genes are expressed in other stages of the *Plasmodium* lifecycle. For example, the ApiAP2 genes *pf11_0091*, *pfd0985w*, *pf11_0442*, and *pff0200c* are expressed in the IDC and also show expression in the early stages of gametocytogenesis [9,10]. Interestingly, *pf13_0026*, *pf11_0091*, *pff0200c*, and *pf11_0442* are associated with the DOZI-associated translational repression complex identified in female gametocytes (we did not identify a motif for PFD0200c which is also repressed by DOZI) [11,12]. Of all the AP2 domains for which we found a motif, only two ApiAP2 genes (*pfl1085w* and *pf13_0026*) are not expressed in the IDC and thus likely function exclusively in one of the other stages of the *P. falciparum* lifecycle.

Motifs bound by multiple ApiAP2 factors

To rigorously evaluate the DNA sequence similarities between motifs, we used the STAMP tool [13] to compare the PWMs for all of our motifs to one another and assign an e-value reflecting how similar two motifs are (Table S1). This analysis identified PWMs from four different AP2 domains to be nearly identical to one another, with a consensus motif of CACACA. Three of the ApiAP2 genes that bind this sequence element, *pfd0985w*, *mal8P1.153*, and *pf14_0533*, are all maximally expressed during the late trophozoite/ early schizont stage of the IDC (Figure S13A). In addition to their similar expression profiles, these three proteins share a number of conserved

residues in the predicted DNA-binding region of the AP2 domain (52% similarity in β-sheet region; Figure S13B). A phylogenetic tree using the β-sheet region of the AP2 domains shows that these three AP2 domains share more homology with each other than with other AP2 domains (Figure S13C). The fourth AP2 domain that binds the CACACA motif is not expressed during the IDC and has a diverged domain sequence (Figure S13B and C), suggesting that primary amino acid sequence alone cannot be used to determine DNA binding specificities.

A second sequence element identified for two different AP2 domains is the GTGCAC motif, which is bound by both PF10_0075_D3 and PFF0200c_DLD (Table S1). PFF0200c_DLD has been shown to interact with two copies of this sequence in the SPE2 motif upstream of *upsB var* genes, but does not bind to other occurrences of this motif throughout the genome [14], while the function of PF10_0075_D3 is currently unknown. The sequence element bound by PFL1085w also has moderate similarity to the GTGCAC motif. Other AP2 pairs with similarity between their PWMs include PF14_0633 and PF11_0091, and PF13_0267 and PFF0670w_D2 (Table S1) however, unlike the above examples these AP2 domains do not bind identical sequences.

Categorization of secondary motifs

Our secondary motifs fall into several previously defined categories [15] in relationship to the primary motifs for each AP2 domain. Three motifs have variable spacer distances, with nucleotide insertions or deletions within a core set of conserved bases. For example, the primary motif for PF10_0075_D1 (GTCGAC) and one of the secondary motifs for this domain (GTC<u>**GC**</u>GAC) reflect a variable spacer distance (Figure S3). The most common change that we observed in secondary motifs (13 motifs) was one or more nucleotide changes at specific positions, typically at either or both the 5' and 3' ends of the primary motif. It is likely that these changes define a central core of the motif, consisting of nucleotides that are absolutely required for binding. For example, the primary motif for PFF0670w_D2 is eight nucleotides (CT<u>**CTAG**</u>AG) while the secondary motif for this AP2 domain maintains the core four nucleotides with changes at either end (AC<u>**CTAG**</u>GT). This result implies that the central nucleotide positions are more important for binding, while the flanking residues may fine-tune the affinity. For another 8 motifs, we observed specific nucleotide changes within the central core of the DNA sequence, suggesting that some AP2 motifs have non-consecutive nucleotides

that are absolutely required for binding (Figure S3). Most striking was the ability of three ApiAP2 proteins to bind multiple motifs with significantly different sequences, implying a level of flexibility in the mode by which ApiAP2 domains interact with their target sequences (e.g. multiple recognition interfaces or conformational changes). Several of the secondary motifs had enrichment scores that were comparable to the primary motif, implying these sites are bound equally well and likely have significant *in vivo* functions (Figure S3, Dataset S2).

Target gene refinement using perturbation data

Perturbation data are inherently richer in their information content with respect to gene expression, because genes that respond in a similar manner to a perturbation are more likely to be regulated by the same factor. Accordingly, we further refined our target gene predictions using a recently published *P. falciparum* perturbation dataset [16]. This dataset has the potential to highlight specific subsets of target genes that are co-ordinately regulated, which we can exploit to better define activity profiles for the ApiAP2 motifs. The activity profile of each motif was used to identify a refined list of target genes that were both enriched for the motif and also responded in a similar manner to various perturbations across the IDC (Figure S7, Dataset S6). This gives a narrower target gene list for each motif, many of which overlap with the predictions made using the IDC co-expression data, and others that are novel target gene predictions (Table 1, Figure 4, Figure S8). A comparison of the relative mRNA abundance profiles of target genes and the corresponding ApiAP2 factor shows stronger correlations compared to the IDC data, but the overall trend (positive or negative relationship) remains unchanged (data not shown). Functional annotation of the perturbation refined targets generally gave similar results to the IDC predictions; however, for a number of motifs the predicted functions were more specific. For example, one of the predicted functions of MAL8P1.153 target genes using the IDC data includes DNA binding, which has also been previously suggested for this motif [17], and this is narrowed to chromosome organization and biogenesis by including the perturbation data in our analysis. Overall, the major functions possibly regulated by our motifs include key cellular processes such as ribosome biogenesis and translation, as well as processes necessary for parasite survival and entry into the host cell (Table 1).

Target genes in non-blood stages of development

To identify putative target gene sets from other stages, we analyzed gene expression data from *P. falciparum* gametocytes and sporozoites [8,10]. Activity profiles for ApiAP2 motifs in gametocyte data revealed activity for a number of the PBM derived motifs during gametocytogenesis (Figure S10A). However, there is no motif that stands out with high positive or negative activity as in the IDC time course, and in fact many motifs show a steady level of activity throughout the entire two week period of gametocytogenesis (Figure S10A). Overall, it appears that several ApiAP2 proteins are active during gametocytogenesis, but there are no striking correlations between stages of gametocyte development, which is likely due to the limited temporal resolution (daily) of the experiments.

We also looked for motif enrichment in *Plasmodium* zygotes. Two ApiAP2 motifs showed activity during this stage, GCTAGC, which is bound by PF11_0442 and CACACA, which is bound by PF13_0026 (Figure S10A). Interestingly, PF13_0026 is not expressed during the asexual stage of development (Figures 1, Figure S13) and may therefore be functional for parasite development in the mosquito. Again, our computational prediction of target genes based on the PBM derived motifs is in complete agreement with the *in vivo* experimental data for this stage. The *P. berghei* orthologue of PF11_0442 (PB000572.01.0) is synthesized in the female gametocyte, but is translationally repressed until the zygote has been formed [11,12]. Upon formation of the zygote PB000572.01.0 is translated, where it activates genes required for mid-gut invasion of the mosquito [18]. We similarly predict PF11_0442 to be involved in activation of target genes during the zygote stage, including a number of genes required for midgut invasion (Dataset S7).

Activity profiles for our motifs in sporozoites identify PF14_0633, PFD0985w, and PFF0670w as potentially active ApiAP2 proteins during this stage (Figure S10A). This result correlates with *in vivo* data that has identified the *P. berghei* orthologue of PF14_0633 (PB000752.01.0) as essential for formation of sporozoites, and defined the GCATGCA sequence as necessary for regulation of sporozoite target genes [19]. The *P. berghei* orthologue of PFD0985w (PB000863.01.0) has also been linked to the sporozoite stage, first as a target gene regulated by PF14_0633 and second via transcript evidence of the *P. berghei* orthologue in salivary gland

sporozoites [19]. No experimental evidence is yet available to associate PFF0670w with the sporozoite stage.

To identify AP2 motifs that are active during the liver stage, we used data from the rodent malaria species *P. yoelii* [20]. The ApiAP2 proteins are conserved among *Plasmodium spp*. and the AP2 domains are highly similar, suggesting that they are likely to bind the same DNA elements. Activity profiles for our PBM derived motifs in the *P. yoelii* liver stage yielded a number of motifs that are potentially active during this stage (Figure S11). Previous work identifying genes that are upregulated in mosquito salivary gland sporozoites found evidence for *pf13_0235* at this stage [21], suggesting that it may be involved in regulating targets during the liver stage. Supporting this hypothesis, we find the G-box motif bound by PF13_0235_D1 to be positively associated with gene expression at 40 hours post invasion. Included among these target genes are a number of ribosomal proteins, which also show enrichment of G-box motifs during the *P. falciparum* blood stages. However, when we examined the 76 *P. falciparum* orthologs of the 98 liver stage *P. yoelii* G-box target genes, we found only 19 with a conserved G-box motif in their upstream regions. This implies that although AP2 domains are conserved in the *Plasmodium spp.*, binding and target gene regulation may be species-specific.

Methods

Identification of IDC and perturbation co-expressed targets

Determination of stage and condition-dependent target genes for each AP2 was done in two steps. In the first step, we used a statistical modelling approach to predict condition- and stage dependent AP2 activity, based on their motif occurrences and gene expression profiles of these conditions and stages. In the second step, we derive the set of genes that have at least one occurrence of each AP2 motif and that correlate with the predicted activity of the same AP2 for the considered stage or condition. Predicted AP2 activity was determined by quantifying how accurately gene expression can be predicted by the number of AP2 motif occurrences in upstream regions of the same genes. Each motif occurrence was weighted, using the ScanACE score of the occurrence divided by the maximum achievable score for that AP2 motif as weight. Then, for each gene expression array we studied, we fitted a linear regression model between the

log-transformed gene expression values (typically log-ratios between condition/stage and control) and the combined weighted motif counts for all AP2s. In mathematical terms, the model can be written as

$$E_i = \sum_{j=1..m} \beta_j c_{ij} + \beta_0 + \varepsilon_i$$

where E_i is the expression value of gene i and c_{ij} is the weighted motif count of the jth AP2 PWM in the upstream region of gene i. The β_j regression coefficients indicate how much each AP2 contributes to the expression values; in other words, they quantify AP2 activity in the studied condition. β_0 corresponds to the baseline gene- and motif-independent expression level. The ε_i term defines the residual stochastic error in the expression measurement, not accountable by motif activity or baseline expression level. Of note, the general application of this modelling approach to transcriptional regulation is not new and has been reported in several other papers [22-24]. Model fitting, *i.e.* determination of the β_j that best explain the expression values given the motif counts, was performed using the Elastic Net approach implemented in the glmnet library in R [25]. We used α=0.05 and estimated λ using 10-fold cross-validation as described in [25]. The Elastic Net approach has a number of advantages over other approaches (e.g. traditional Ordinary Least Squares (OLS) and LASSO) in that it produces sparse models (where many regression coefficients are set to 0) but also allows for correlated AP2 activities (that is, two AP2s with similar motif counts in each upstream regions will contribute equally to the model, unlike what would happen with OLS or LASSO). Overall model fit was measured as the Pearson correlation between actual expression values and expression values predicted by the fitted linear model. To account for model overfitting, models fits were always compared to fits obtained from the same motif count data but from randomized expression values. The predicted AP2 activities (fitted β_j regression coefficients) were then used to define expression dataset-dependent target genes. In datasets with multiple time points (e.g. the IDC), target genes are defined as genes with at least one occurrence of an AP2 motif and whose expression profile correlates positively with the activity profile of the same AP2 calculated across the same time points. Correlation between expression profiles and activity profiles was quantified using the Pearson correlation, and constrained to be >0.5 and with adjusted p-value <0.01 (corresponding to 1% FDR). In datasets with single condition/stage, target genes were defined as genes with the AP2 motif and whose expression "follows" the predicted AP2 activity (e.g. >2-fold up- or down

regulation depending on the sign of the AP2 β_j regression coefficient). All R scripts and Perl programs used to perform these analyses are available at http://physiology.med.cornell.edu/faculty/elemento/lab/ApiAP2/.

Analysis of nucleosome placement and binding site accessibility

Raw 36bp-long Illumina reads for the FAIRE-seq and MAINE-seq experiments downloaded from the NCBI short read archive and aligned to the *P. falciparum* (release 6.0) genome using the Burrows-Wheeler alignment program [26], with default parameters. Only uniquely mapping reads (XT:A:U in the SAM format) were retained. For each experiment, the number of reads mapping across (i.e. overlapping with) each position (nt) in the *P. falciparum* genome was calculated. This defines a read count profile across the entire genome. Genome-wide short read (36b-long) mappability was also computed using k-mer counting tools in GenomeTools framework [27,28] so as to determine nucleotides in the *P. falciparum* genome that lie in regions that are not unique in the genome, and therefore where a lower read count is expected compared to unique regions. All motif occurrences in these regions were ignored in subsequent analyses. For each FAIRE-seq and MAINE-seq datasets, we then determined the genome-wide 25th read count percentile, i.e. the read count value such that 75% of read counts at mappable nucleotides are below that threshold. Only nucleotide positions residing in mappable regions were used for this calculation. MAINE-seq read counts above the 25th percentile and FAIRE-seq regions above the 25th percentile were considered to be nucleosome-free regions/open chromatin. For each 6bp-long AP2 motif occurrence, we then calculated the average read count in the FAIRE-seq and MAINE-seq experiments, for all time points. These read counts were compared to the 25th percentiles thresholds described above to determine whether they fall in nucleosome-free regions/open chromatin or not.

References

1. Berger MF, Bulyk ML (2009) Universal protein-binding microarrays for the comprehensive characterization of the DNA-binding specificities of transcription factors. Nat Protoc 4: 393-411.
2. Berger MF, Bulyk ML (2006) Protein binding microarrays (PBMs) for rapid, high-throughput characterization of the sequence specificities of DNA binding proteins. Methods Mol Biol 338: 245-260.

3. Berger MF, Philippakis AA, Qureshi AM, He FS, Estep PW, 3rd, et al. (2006) Compact, universal DNA microarrays to comprehensively determine transcription-factor binding site specificities. Nat Biotechnol 24: 1429-1435.
4. Nole-Wilson S, Krizek BA (2000) DNA binding properties of the *Arabidopsis* floral development protein AINTEGUMENTA. Nucleic Acids Res 28: 4076-4082.
5. Cole C, Barber JD, Barton GJ (2008) The Jpred 3 secondary structure prediction server. Nucleic Acids Res 36: W197-201.
6. Hughes JD, Estep PW, Tavazoie S, Church GM (2000) Computational identification of *cis*-regulatory elements associated with groups of functionally related genes in *Saccharomyces cerevisiae*. J Mol Biol 296: 1205-1214.
7. Bozdech Z, Llinas M, Pulliam BL, Wong ED, Zhu J, et al. (2003) The transcriptome of the intraerythrocytic developmental cycle of *Plasmodium falciparum*. PLoS Biol 1: E5.
8. Le Roch KG, Zhou Y, Blair PL, Grainger M, Moch JK, et al. (2003) Discovery of gene function by expression profiling of the malaria parasite life cycle. Science 301: 1503-1508.
9. Silvestrini F, Bozdech Z, Lanfrancotti A, Di Giulio E, Bultrini E, et al. (2005) Genome-wide identification of genes upregulated at the onset of gametocytogenesis in *Plasmodium falciparum*. Mol Biochem Parasitol 143: 100-110.
10. Young JA, Fivelman QL, Blair PL, de la Vega P, Le Roch KG, et al. (2005) The *Plasmodium falciparum* sexual development transcriptome: a microarray analysis using ontology-based pattern identification. Mol Biochem Parasitol 143: 67-79.
11. Mair GR, Braks JA, Garver LS, Wiegant JC, Hall N, et al. (2006) Regulation of sexual development of *Plasmodium* by translational repression. Science 313: 667-669.
12. Mair GR, Lasonder E, Garver LS, Franke-Fayard BM, Carret CK, et al. (2010) Universal features of post-transcriptional gene regulation are critical for *Plasmodium* zygote development. PLoS Pathog 6: e1000767.
13. Mahony S, Benos PV (2007) STAMP: a web tool for exploring DNA-binding motif similarities. Nucleic Acids Res 35: W253-258.
14. Flueck C, Bartfai R, Neiderwieser I, Witmer K, Alako BTF, et al. (2010) A Major Role for the *Plasmodium falciparum* ApiAP2 Protein PFSIP2 in Chromosome End Biology. PLoS Pathog 6: e1000784.
15. Badis G, Berger MF, Philippakis AA, Talukder S, Gehrke AR, et al. (2009) Diversity and complexity in DNA recognition by transcription factors. Science 324: 1720-1723.
16. Hu G, Cabrera A, Kono M, Mok S, Chaal BK, et al. (2010) Transcriptional profiling of growth perturbations of the human malaria parasite *Plasmodium falciparum*. Nat Biotechnol 28: 91-98.
17. Young JA, Johnson JR, Benner C, Yan SF, Chen K, et al. (2008) In silico discovery of transcription regulatory elements in *Plasmodium falciparum*. BMC Genomics 9: 70.
18. Yuda M, Iwanaga S, Shigenobu S, Mair GR, Janse CJ, et al. (2009) Identification of a transcription factor in the mosquito-invasive stage of malaria parasites. Mol Microbiol 71: 1402-1414.
19. Yuda M, Iwanaga S, Shigenobu S, Kato T, Kaneko I (2010) Transcription Factor AP2-Sp and its Target Genes in Malarial Sporozoites. Mol Microbiol 75: 854-863.
20. Tarun AS, Peng X, Dumpit RF, Ogata Y, Silva-Rivera H, et al. (2008) A combined transcriptome and proteome survey of malaria parasite liver stages. Proc Natl Acad Sci U S A 105: 305-310.

21. Mikolajczak SA, Silva-Rivera H, Peng X, Tarun AS, Camargo N, et al. (2008) Distinct malaria parasite sporozoites reveal transcriptional changes that cause differential tissue infection competence in the mosquito vector and mammalian host. Mol Cell Biol 28: 6196-6207.
22. Bussemaker HJ, Li H, Siggia ED (2001) Regulatory element detection using correlation with expression. Nat Genet 27: 167-171.
23. Foat BC, Houshmandi SS, Olivas WM, Bussemaker HJ (2005) Profiling condition-specific, genome-wide regulation of mRNA stability in yeast. Proc Natl Acad Sci U S A 102: 17675-17680.
24. Foat BC, Tepper RG, Bussemaker HJ (2008) TransfactomeDB: a resource for exploring the nucleotide sequence specificity and condition-specific regulatory activity of trans-acting factors. Nucleic Acids Res 36: D125-131.
25. Zou H, Hastie T (2005) Regularization and variable selection via the elastic net. J R Statist Soc B 67: 301-320.
26. Li H, Durbin R (2009) Fast and accurate short read alignment with Burrows-Wheeler transform. Bioinformatics 25: 1754-1760.
27. Lichtenberg J, Jacox E, Welch JD, Kurz K, Liang X, et al. (2009) Word-based characterization of promoters involved in human DNA repair pathways. BMC Genomics 10 Suppl 1: S18.
28. Lichtenberg J, Yilmaz A, Welch JD, Kurz K, Liang X, et al. (2009) The word landscape of the non-coding segments of the *Arabidopsis thaliana* genome. BMC Genomics 10: 463.
29. Bozdech Z, Mok S, Hu G, Imwong M, Jaidee A, et al. (2008) The transcriptome of *Plasmodium vivax* reveals divergence and diversity of transcriptional regulation in malaria parasites. Proc Natl Acad Sci U S A 105: 16290-16295.
