# Supplementary figures and images for: Identification and Genome-Wide Prediction of DNA Binding Specificities for the ApiAP2 Family of Regulators from the Malaria Parasite
Source: PLoS Pathog. 2010 Oct 28;6(10):e1001165. doi: 10.1371/journal.ppat.1001165 (PMC2965767; doi:10.1371/journal.ppat.1001165)

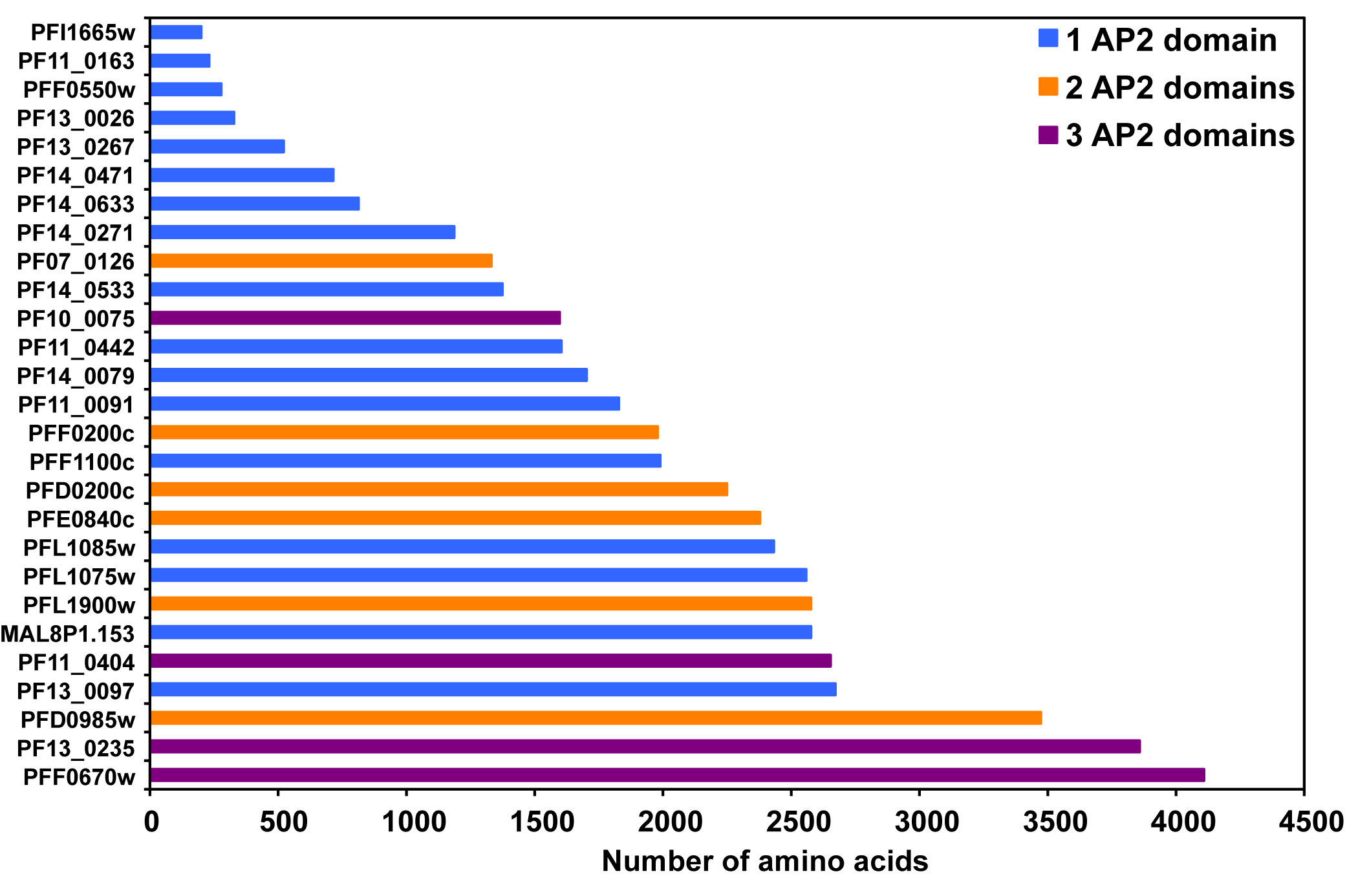

Supplement: Figure S1 — Size distribution of ApiAP2 proteins. Proteins range in size from 200 amino acids to over 4000, with the four smallest ApiAP2 proteins having less than 500 amino acids. Bars are colour coded based on the number of AP2 domains in each protein. (0.38 MB TIF) [file ppat.1001165.s002.tif]

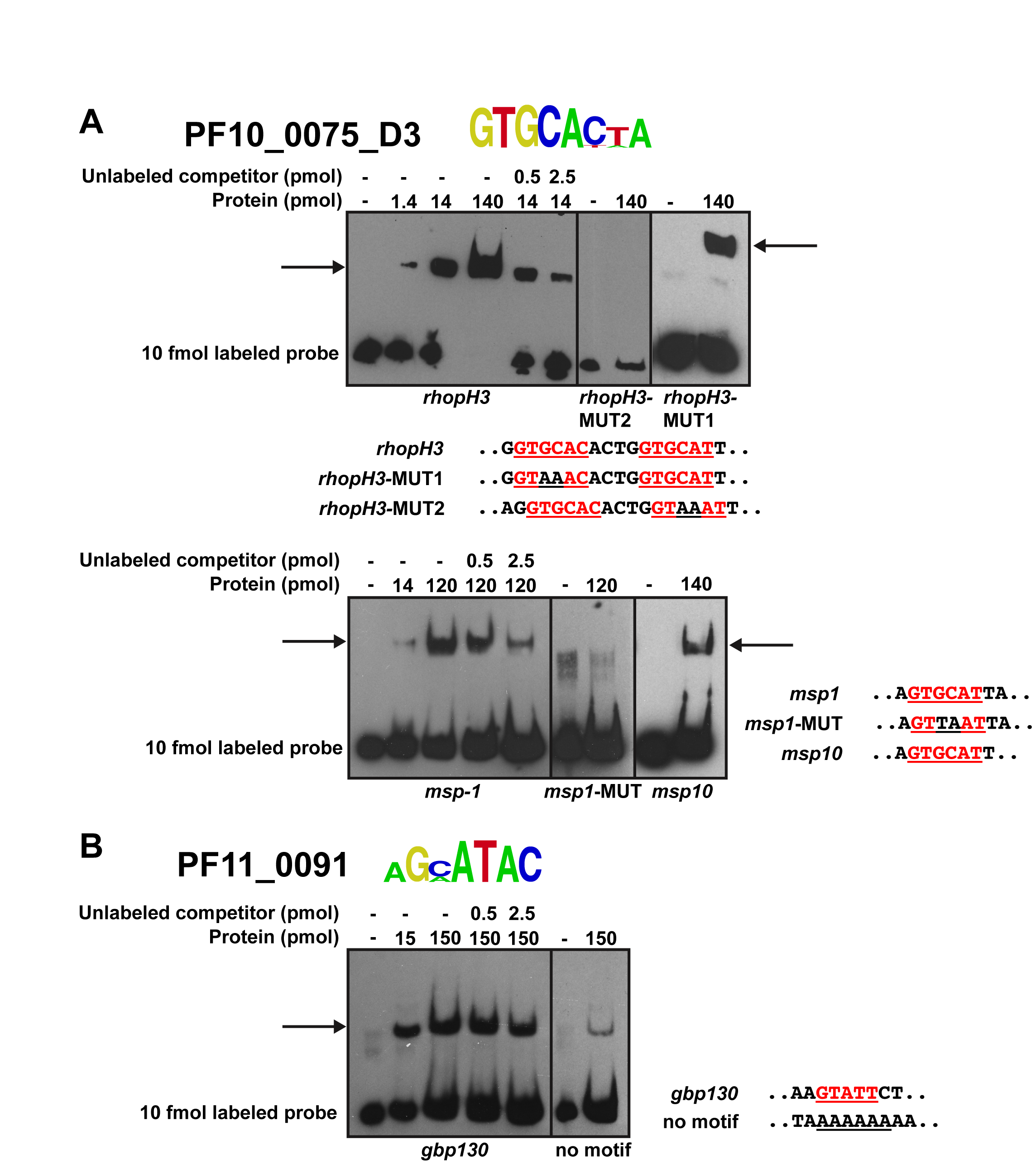

Supplement: Figure S4 — PF10_0075_D3 and PF11_0091 bind Plasmodium regulatory motifs. A) Partial sequences of the EMSA probes from the upstream sequences of rhopH3, msp1 and msp10. The PF10_0075_D3 motif is underlined in red and mutations are underlined in black. EMSAs using these probes illustrate the ability of PF10_0075_D3 to specifically bind the target sequences (shifted complexes are denoted with an arrow). No binding was observed with an unrelated oligonucleotide. Probes are biotin labeled and all competitors are unlabeled. B) EMSA using the gbp130 upstream sequence demonstrates that PF11_0091 binds to this sequence, but not to a non-specific probe. Sequences are indicated as in (A). (1.84 MB TIF) [file ppat.1001165.s005.tif]

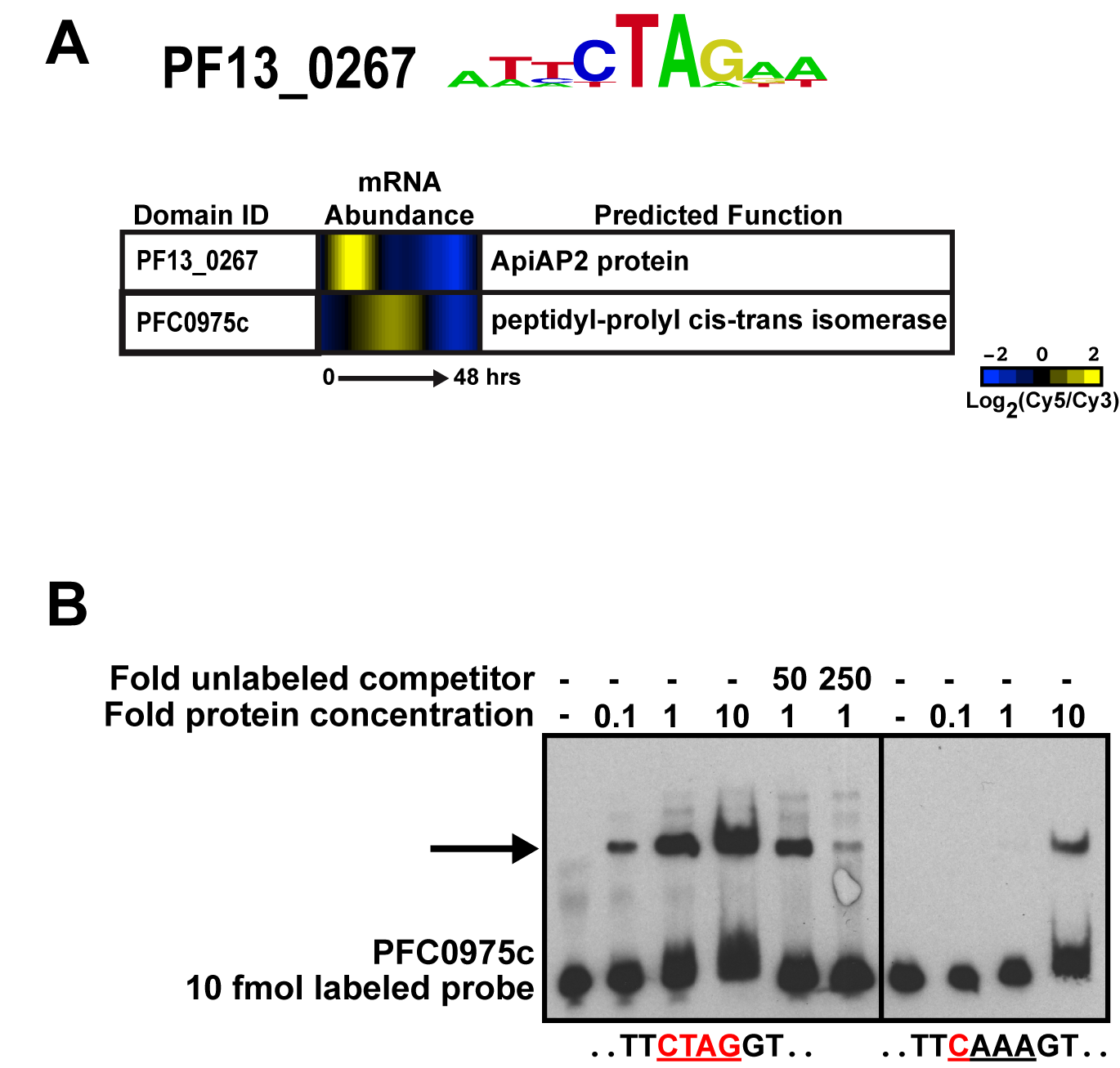

Supplement: Figure S5 — PF13_0267 binds to a sequence upstream of a ScanACE predicted target gene. A) Transcript abundance profiles of pf13_0267 and a predicted target gene, pfc0975c, show similar timing [7]. B) EMSA using the upstream sequence of pfc0975c, a putative target of PF13_0267. Biotinylated probe is specifically shifted with increasing amounts of the purified protein (designated by the arrow) and this shift is competed with unlabeled probe DNA. No shift is observed with an unrelated oligonucleotide (data not shown). Partial probe sequences are shown below the gel, with the PF13_0267 target motif underlined in red and mutations of the motif underlined in black. (0.63 MB TIF) [file ppat.1001165.s006.tif]

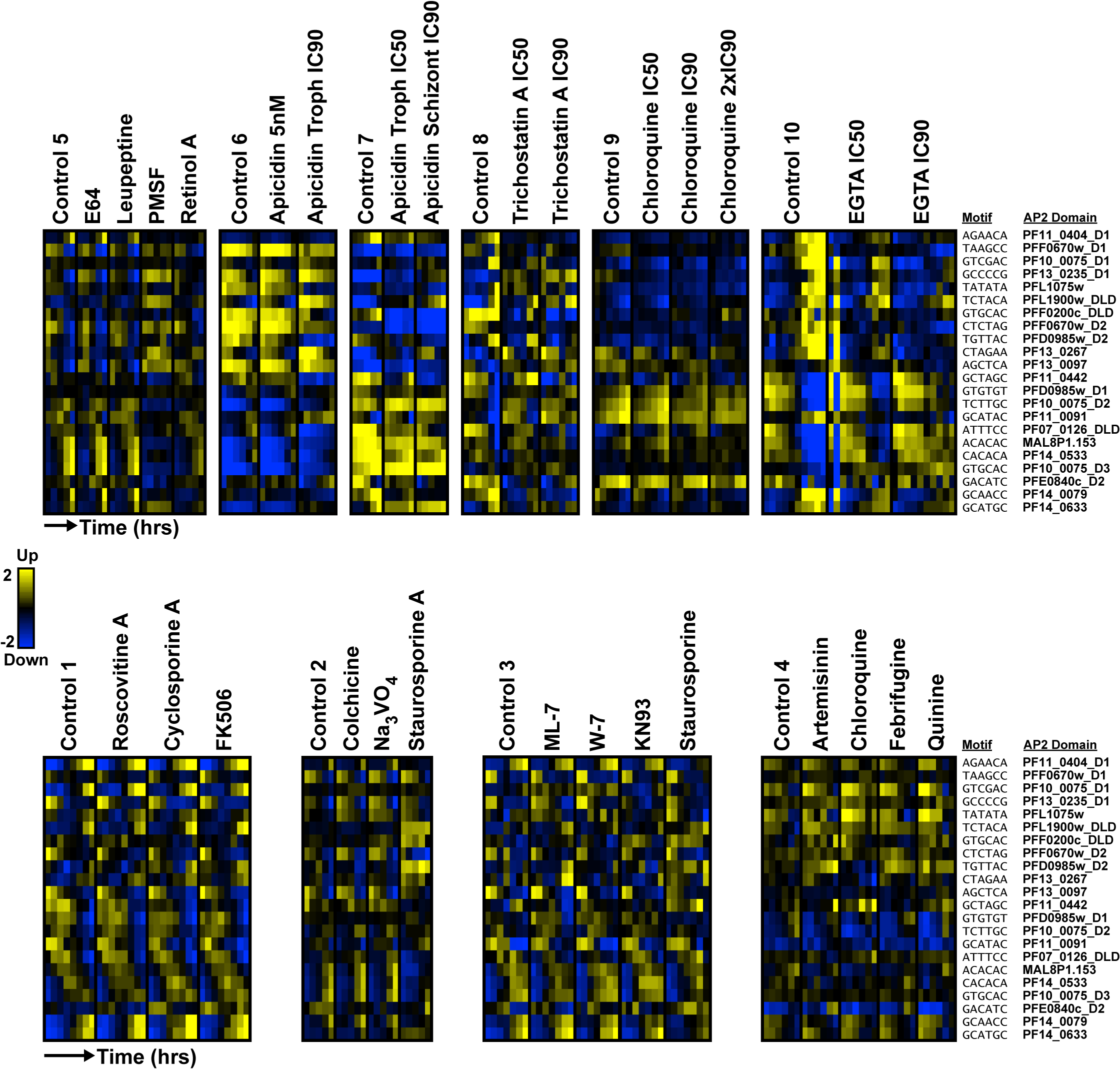

Supplement: Figure S7 — Activity profiles for AP2 motifs using perturbation data. To refine our list of target genes we defined activity profiles for each motif using the IDC perturbation data [16]. Activity profiles are grouped by drug treatments and their corresponding controls. Each row represents the motif activity profiles and timepoints are from left to right within each treatment. Specific details for each perturbation experiment can be found in [16]. (2.25 MB TIF) [file ppat.1001165.s008.tif]

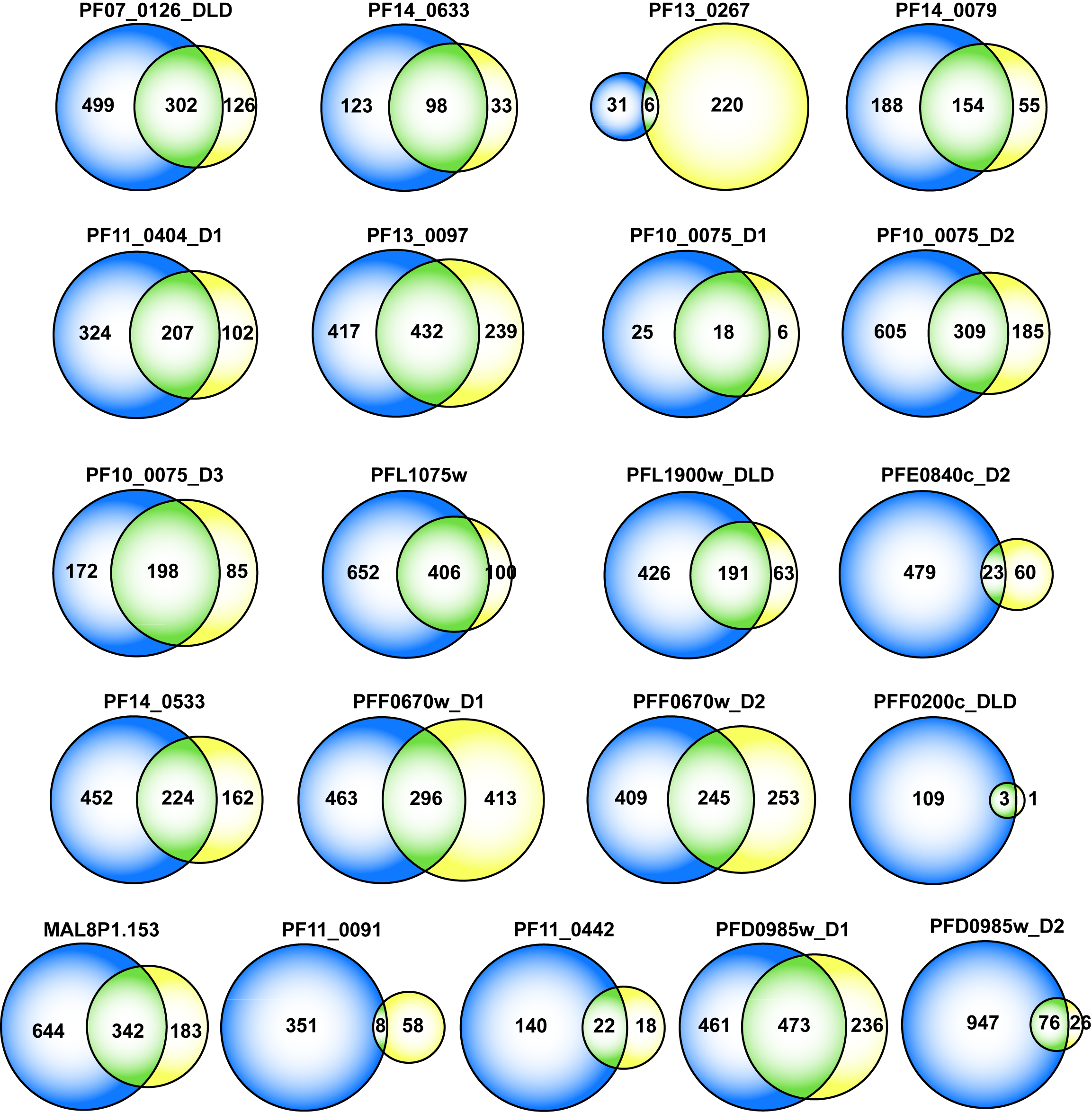

Supplement: Figure S8 — Overlap between predicted IDC and perturbation co-expressed targets. Blue circles represent the predicted IDC co-expressed targets and the yellow shaded circles are the perturbation co-expressed targets. The overlap between the two gene lists is shown in green. The numbers indicate the number of unique gene IDs in each dataset. The AP2 domain that binds to the corresponding motif is listed above each Venn diagram. (4.62 MB TIF) [file ppat.1001165.s009.tif]

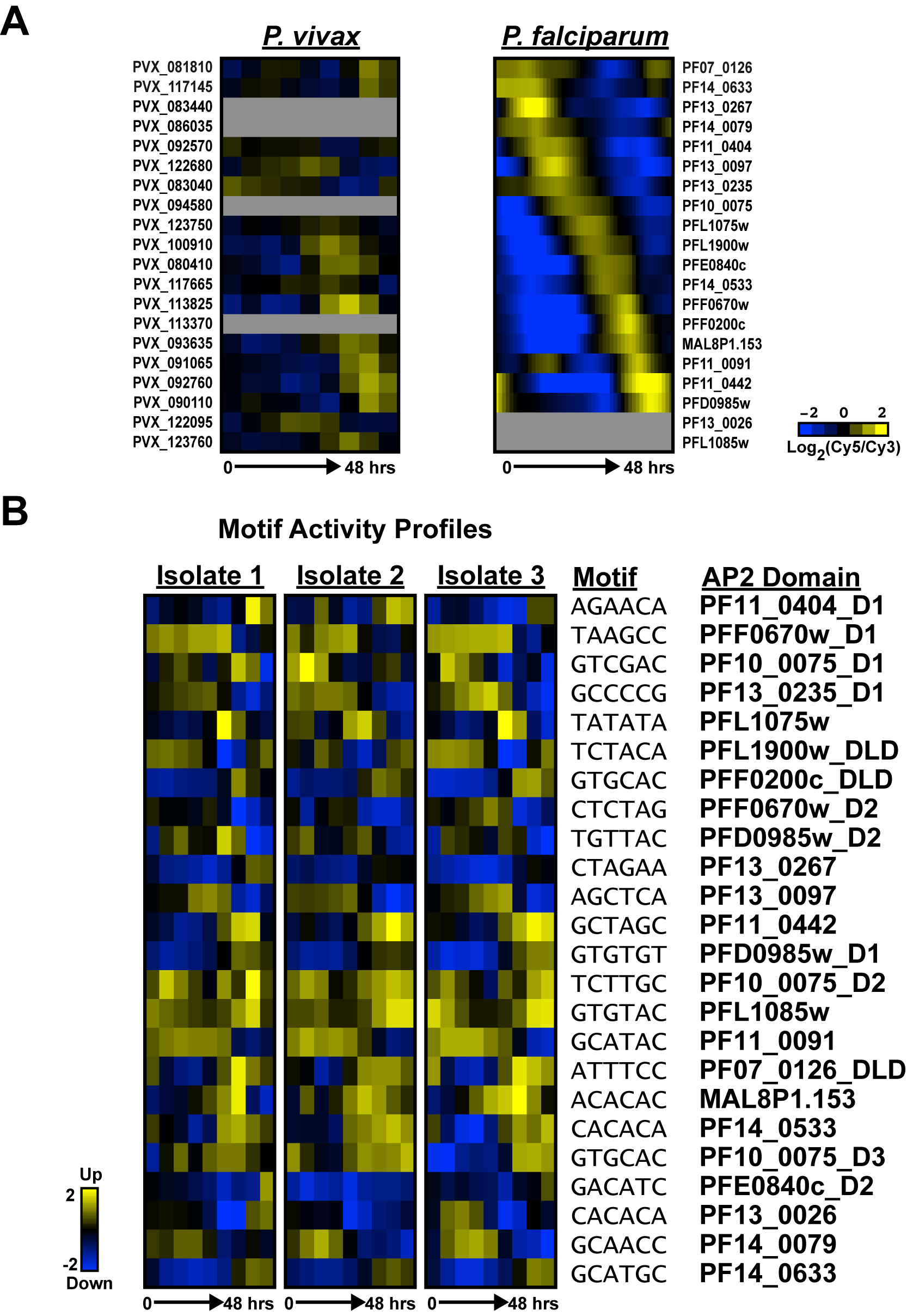

Supplement: Figure S9 — ApiAP2 IDC expression and activity profiles for the AP2 motifs in P. vivax samples. A) A comparison of IDC mRNA abundance profiles for the P. falciparum ApiAP2 proteins [7] with motif data and their P. vivax orthologs [29]. Expression is similar between the two species. Gray indicates data not available. B) To compare target genes for each motif in P. falciparum and P. vivax, activity profiles were defined using three P. vivax isolates [29]. The columns in the heat map represent the nine timepoints and rows are the motif activity profiles. ApiAP2 proteins are ordered as in Figure 4. (1.85 MB TIF) [file ppat.1001165.s010.tif]

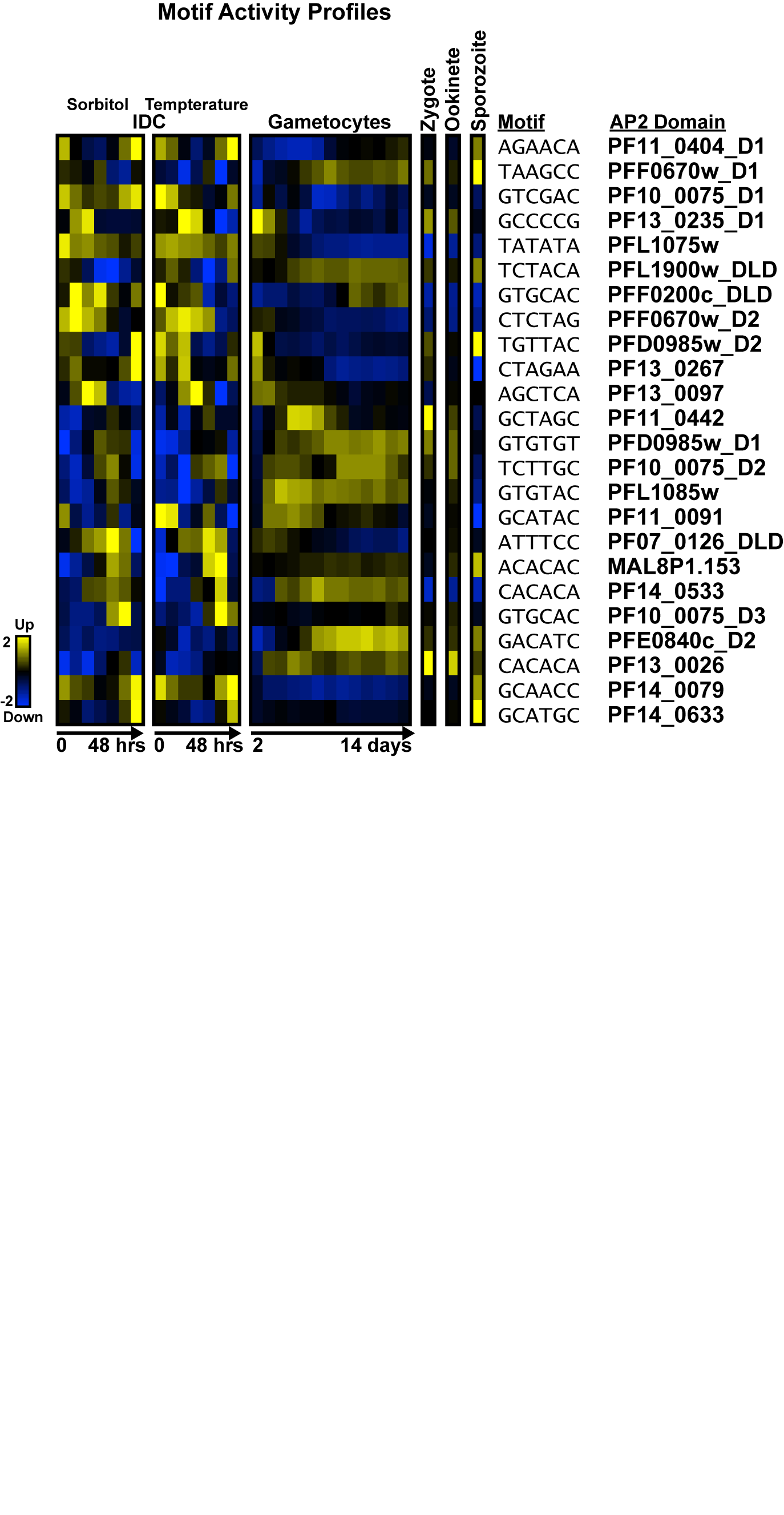

Supplement: Figure S10 — Activity profiles for AP2 motifs in different stages of the P. falciparum lifecycle and target gene predictions. To identify motifs that function in different stages we used data from across the lifecycle [8], [10]. Motif activity profiles for the IDC are in duplicate, using either sorbitol or temperature synchronized parasites. Data for gametocyte expression is from a 14 day experiment and zygotes, ookinetes, and sporozoites represent a single timepoint. Each row is the activity profile for an AP2 motif. (1.04 MB TIF) [file ppat.1001165.s011.tif]

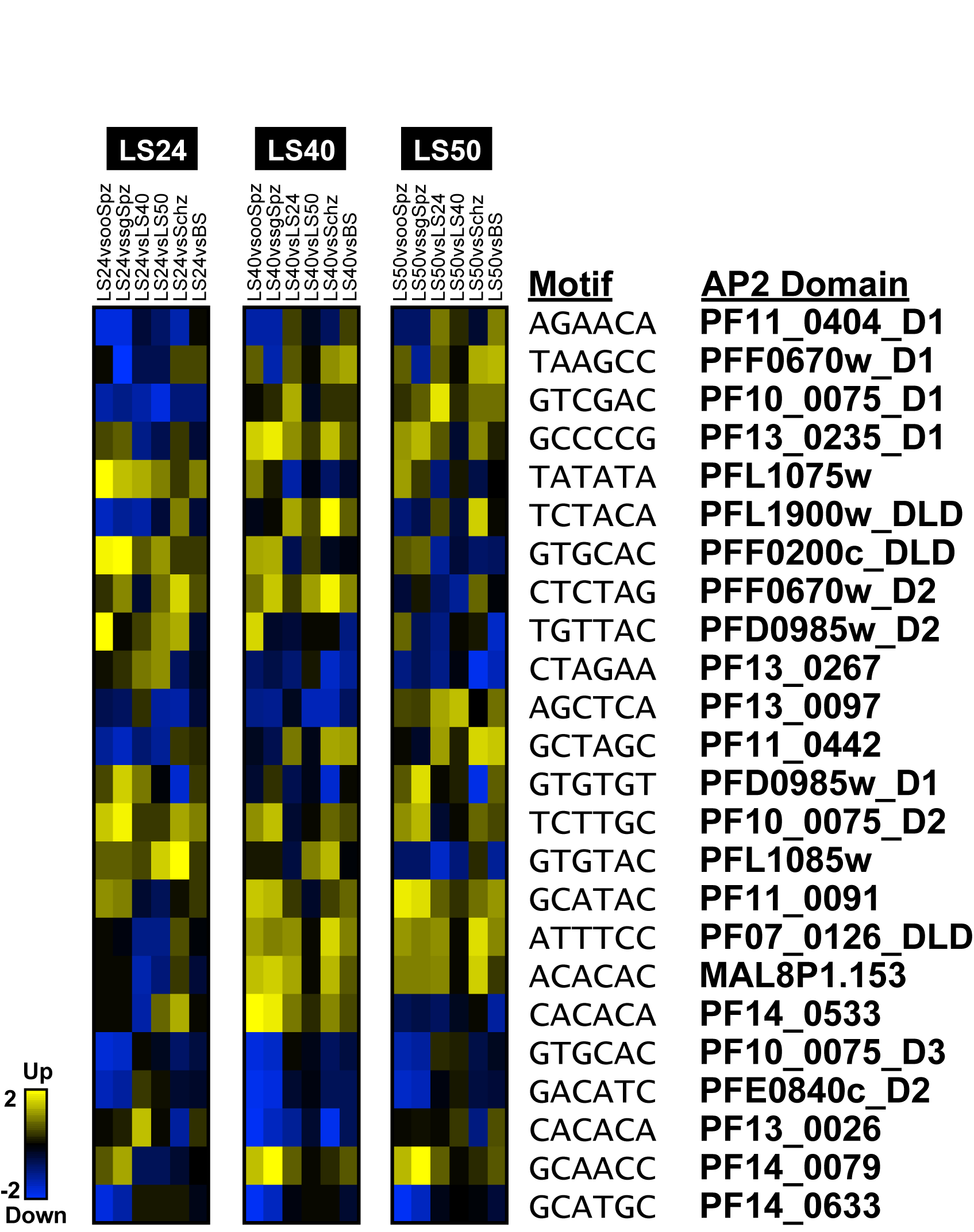

Supplement: Figure S11 — AP2 motif activity profiles during the P. yoelii liver stage. Motifs for the P. falciparum ApiAP2 proteins were used to establish activity profiles in the P. yoelii liver stage (LS) [20]. Columns are grouped based on the hour post invasion in the liver (24, 40, or 50 hours). At each timepoint LS samples were compared to a range of samples: mosquito oocyst sporozoites (ooSpz), mosquito salivary gland sporozoites (sgSpz), to alternate LS timepoints, and to blood stage schizonts (sSchz) and mixed blood stage samples (BS). ApiAP2 proteins are ordered as in Figure 4. (1.26 MB TIF) [file ppat.1001165.s012.tif]

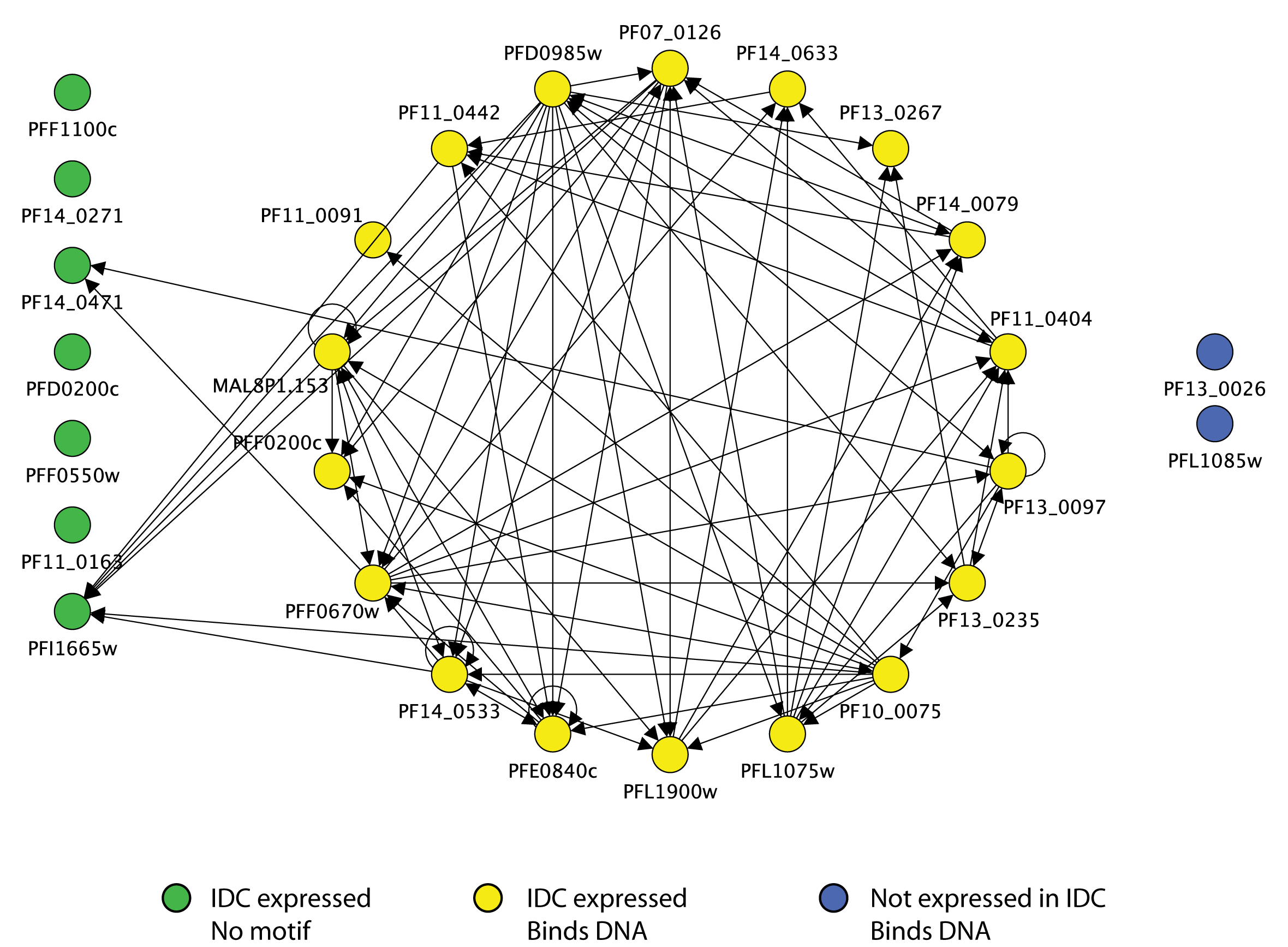

Supplement: Figure S12 — P. falciparum IDC ApiAP2 regulatory network. ApiAP2 genes are targets of other ApiAP2 factors. ApiAP2 genes are arranged in order of expression during the IDC in a clockwise manner starting at PF07_0126. Arrows pointing away from an ApiAP2 gene indicate that it potentially regulates the target factor by binding to motifs in the target upstream region. ApiAP2 genes coloured in green are expressed during the IDC, but did not exhibit DNA binding specificity on the PBMs. Genes in yellow are expressed during the IDC and bind to specific DNA sequences and genes in blue also bind DNA, but are not expressed during the IDC. (0.67 MB TIF) [file ppat.1001165.s013.tif]

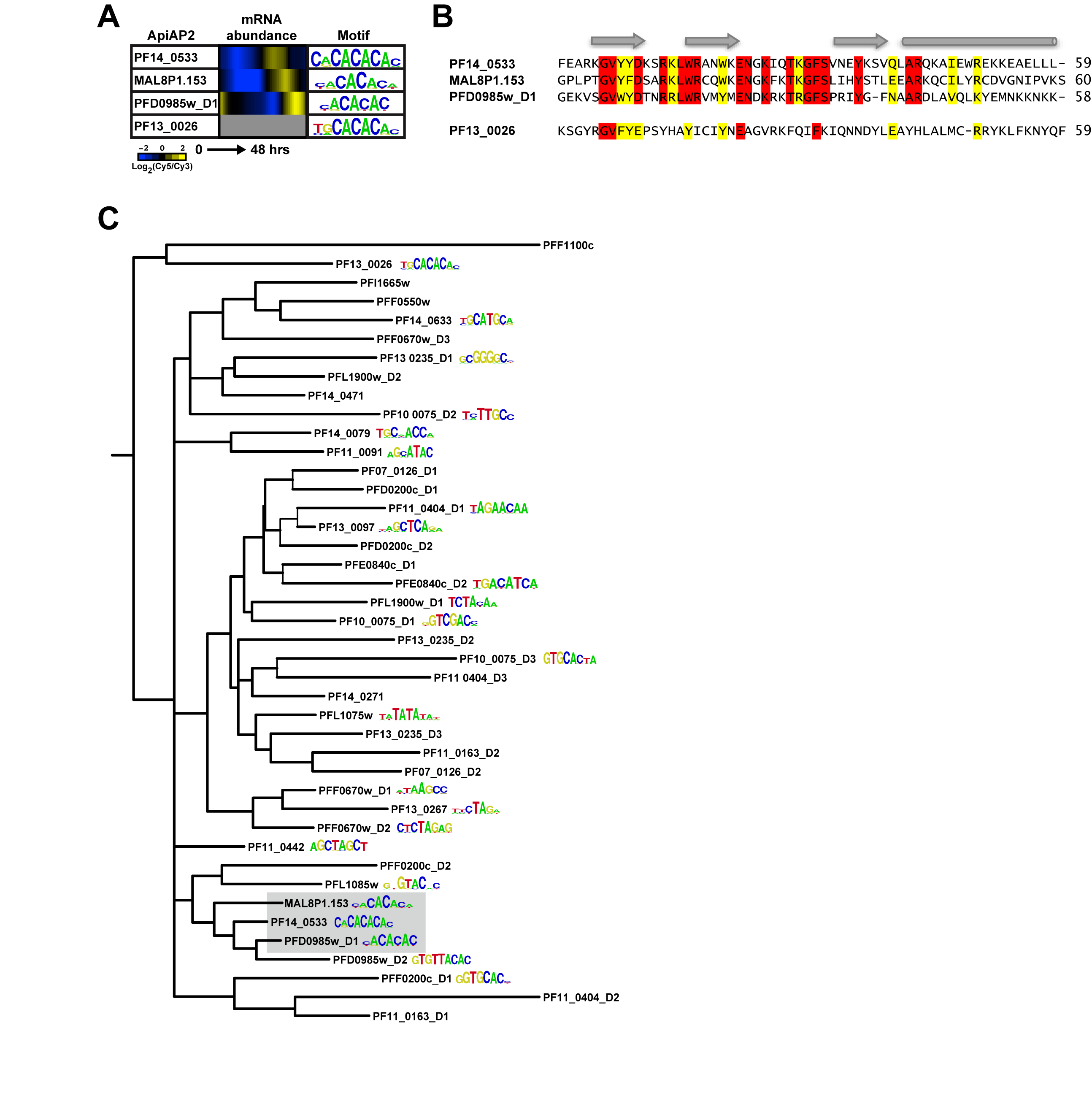

Supplement: Figure S13 — ApiAP2 proteins that bind the CACACA motif. A) Three of the ApiAP2 factors that bind the CACACA motif are expressed in the late stages of the IDC as shown by mRNA abundance profiles [7]. B) An alignment (performed using ClustalW; www.ebi.ac.uk/clustalw) of the AP2 domains for these three factors demonstrates a high level of similarity (52%) in the predicted β-sheets (gray arrows above the alignment); which likely contain the DNA binding residues. Identical residues are highlighted in red and similar residues in yellow. Secondary structure predictions were made using Jpred3 [5]. Addition of PF13_0026 to the alignment, the one CACACA-binding factor that is not expressed in the IDC, shows that the sequence of this AP2 domain is more divergent. (C) Phylogenetic tree of the predicted β-sheet regions of the AP2 domains. The tree demonstrates that the three IDC expressed ApiAP2 factors that bind the CACACA motif are more similar to one another than to any other AP2 domain. The tree was made using PhyML, using maximum likelihood with a LG protein evolution model. (0.84 MB TIF) [file ppat.1001165.s014.tif]
