# Supplementary material for: Identification and Genome-Wide Prediction of DNA Binding Specificities for the ApiAP2 Family of Regulators from the Malaria Parasite
Source: PLoS Pathog. 2010 Oct 28;6(10):e1001165. doi: 10.1371/journal.ppat.1001165 (PMC2965767; doi:10.1371/journal.ppat.1001165)
Supplement: Figure S2 — ApiAP2 domains tested on PBMs. ApiAP2 proteins are listed in order of size. Domains were cloned into pGEX-4T1 to produce N-terminal GST fusions. Proteins were expressed and purified from E. coli and tested in duplicate on protein binding microarrays (PBMs). D1 indicates the AP2 domain closest to the N-terminus; D2 and D3 are the AP2 domains following D1 going from the N- to C-terminus of the protein; DLD indicates two domains and a short linker region (Domain - Linker - Domain); ext at the end of the domain number indicates an extension of the original cloned domain at either or both of the N- and C-termini. Enrichment scores above 0.450 were considered significant, and no result indicates an E-score below this cut-off. PFL1900w_DLD has a poly-asparagine tract that increases its linker length by 39 amino acids. This expanded linker is absent in the P. berghei orthologue (PB000218.00.0) of PFL1900w, while the AP2 domain sequences are 99% identical. To test the effect of the PFL1900w expanded linker on DNA binding we generated a GST fusion DLD construct for the shorter P. berghei orthologue. Both constructs exhibited identical DNA binding specificity. (0.26 MB PDF) [file ppat.1001165.s003.pdf]

ApiAP2		Amino acids cloned	Motif	Enrichment score
PFI1665w	Full length	1-200	No result	
	D1	109-166	No result	
PF11_0163	Full length	1-231	No result	
	D1	114-172	No result	
	D2	181-231	No result	
	DLD	114-231	Did not test	
PFF0550w	Full length	1-278	No result	
	D1	168-226	No result	
PF13_0026	Full length	1-328	No result	
	D1	255-313	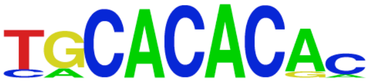	0.495
PF13_0267	D1	400-521	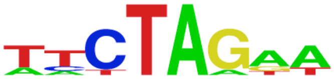	0.479
PF14_0471	D1	46-103	No result	
PF14_0633	D1	63-123	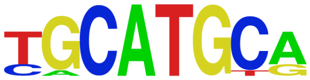	0.500
PF14_0271	D1	51-109	No result	
PF07_0126	D1	993-1051	No result	
	D1ext	944-1051	No result	
	D2	1076-1129	No result	
	D2ext	1076-1160	No result	
	DLD	993-1129	No result	
	DLDext	944-1160	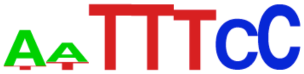	0.467
PF14_0533	D1	1316-1374	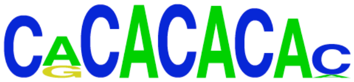	0.500
PF10_0075	D1	522-580	No result	
	D1ext	483-586	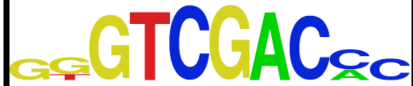	0.495
	D2	771-829	No result	
	D2ext	771-862	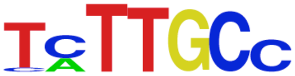	0.468
	D3	941-1002	No result	
	D3ext	910-1027	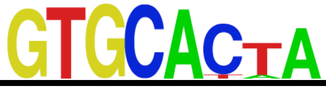	0.496
PF11_0442	D1	1364-1420	No result	
	D1ext	1326-1428	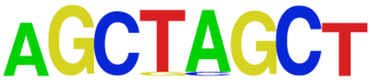	0.478

ApiAP2		Amino acids cloned	Motif	Enrichment score
PF14_0079	D1	1597-1660	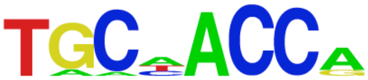	0.487
PF11_0091	D1	1486-1548	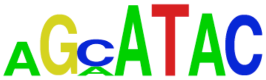	0.472
PFF0200c	D1	177-235	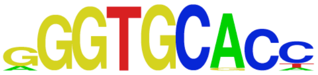	0.500
	D2	253-312	No result	
	D2ext	253-355	No result	
	DLD	177-312	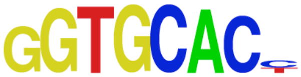	0.481
PFF1100c	D1	871-929	No result	
	D1ext-1	806-927	Did not express	
	D1ext-2	718-927	No result	
PFD0200c	D1	235-294	No result	
	D1ext	209-294	No result	
	D2	1149-1203	No result	
PFE0840c	D1	950-1008	No result	
	D1ext	928-1022	No result	
	D2	1802-1861	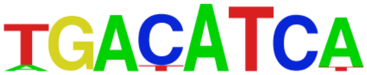	0.472
PFL1085w	D1	2157-2217	No result	
	D1ext	2123-2245	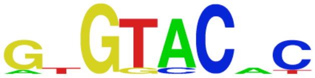	0.490
PFL1075w	D1	1871-1929	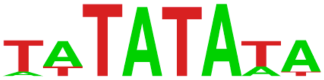	0.499
PFL1900w	D1	2149-2207	No result	
	D1ext	2104-2218	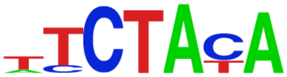	0.460
	D2	2288-2347	No result	
	DLD	2149-2347	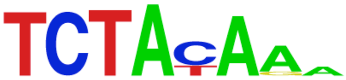	0.468
PB000218.00.0	DLD	948-1107	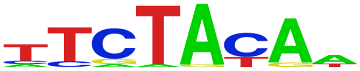	0.458
MAL8P1.153	D1	2520-2577	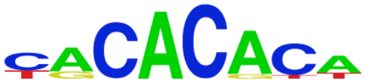	0.477
PF11_0404	D1	285-343	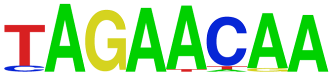	0.485
	D2	377-435	No result	

ApiAP2		Amino acids cloned	Motif	Enrichment score
PF11_0404	D2ext	377-453	No result	
	DLD	285-435	No result	
	DLDext	285-453	No result	
	D3	1828-1882	No result	
	D3ext	1791-1902	No result	
PF13_0097	D1	1051-1109	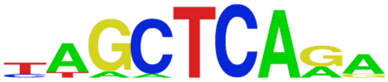	0.489
PFD0985w	D1	1956-2015	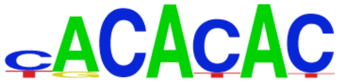	0.497
	D2	3267-3325	No result	
	D2ext	3258-3362	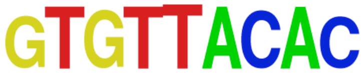	0.496
PF13_0235	D1	2361-2419	No result	
	D1ext	2339-2440	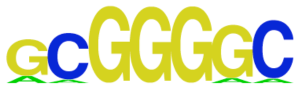	0.494
	D2	3065-3124	No result	
	D2ext	3055-3147	No result	
	D3	3788-3846	No result	
	D3ext	3709-3858	No result	
PFF0670w	D1	3083-3141	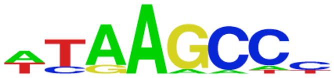	0.465
	D2	3672-3730	No result	
	D2ext	3648-3730	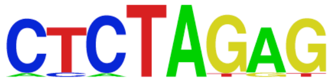	0.495
	D3	4030-4092	No result	
	D3ext	3999-4109	No result	
