# Supplementary material for: Identification and Genome-Wide Prediction of DNA Binding Specificities for the ApiAP2 Family of Regulators from the Malaria Parasite
Source: PLoS Pathog. 2010 Oct 28;6(10):e1001165. doi: 10.1371/journal.ppat.1001165 (PMC2965767; doi:10.1371/journal.ppat.1001165)
Supplement: Figure S3 — AP2 domain secondary motifs. Secondary motifs found for ApiAP2 proteins and their associated enrichment scores. E-scores greater than 0.450 were considered significant. The final column lists the relationship of the secondary motif to the primary motif using the following descriptions: end modification is a change in nucleotide specificity at either or both the 5′ and 3′ ends of the motif, alternate recognition interface is a motif that is unrelated to the primary motif, variable spacer distance is an insertion or deletion in the middle of the motif, and core change is a change in nucleotide specificity in the middle of the motif. (0.26 MB PDF) [file ppat.1001165.s004.pdf]

ApiAP2	Secondary Motif	Enrichment Score	Relationship to 1° Motif
PF14_0633	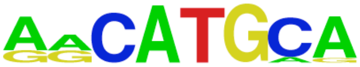	0.490	End modification
PF14_0533	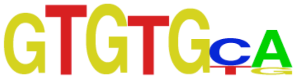	0.487	End modification
PF10_0075_D1	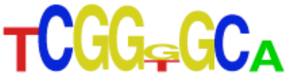	0.488	Alternate recognition interface
	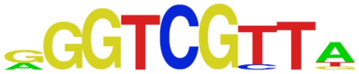	0.482	End modification
	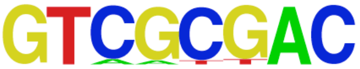	0.481	Variable spacer distance
	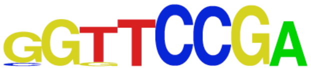	0.471	Alternate recognition interface
PF10_0075_D3	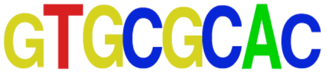	0.493	Variable spacer distance
	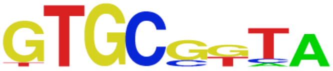	0.471	Core change
	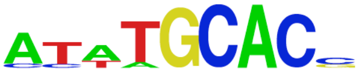	0.459	End modification
	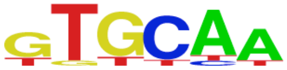	0.454	End modification
PFF0200c_D1	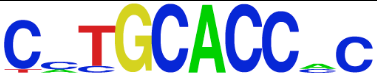	0.495	End modification
	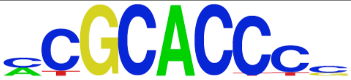	0.494	End modification
	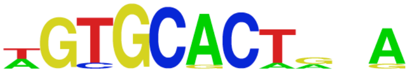	0.497	End modification
PFL1085w	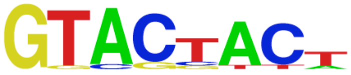	0.469	Core change
	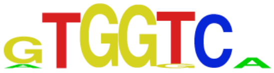	0.462	Alternate recognition interface
PFL1075w	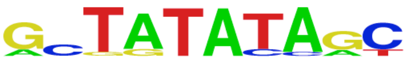	0.494	End modification
	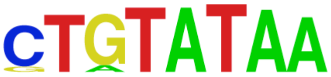	0.488	Core change
PFL1900w_DLD	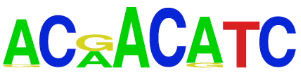	0.472	Alternate recognition interface

ApiAP2	Secondary Motif	Enrichment Score	Relationship to 1° Motif
PFL1900w_DLD	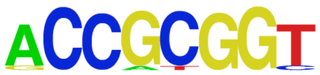	0.474	Alternate recognition interface
PF11_0404_D1	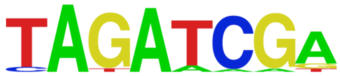	0.460	Core change
PF13_0097	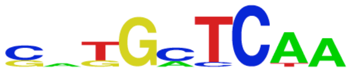	0.476	End modification
	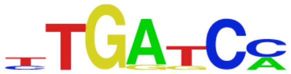	0.476	Core change
	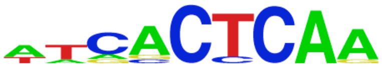	0.481	End modification
PFD0985w_D2	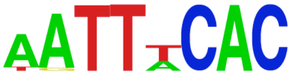	0.495	End modification
	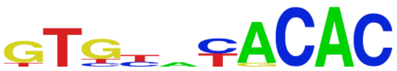	0.494	Variable spacer distance
	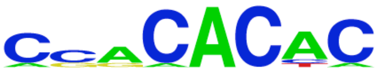	0.493	Core change
PF13_0235_D1	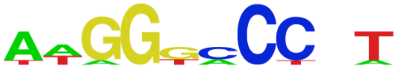	0.482	Core change
PFF0670w_D2	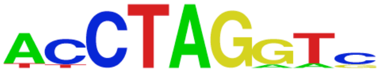	0.488	End modification
	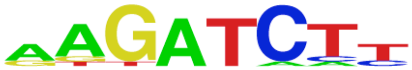	0.456	Core change
PF13_0267	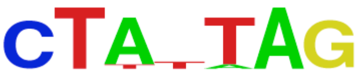	0.475	Variable spacer distance
	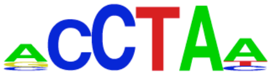	0.473	End modification
