# Supplementary material for: Identification and Genome-Wide Prediction of DNA Binding Specificities for the ApiAP2 Family of Regulators from the Malaria Parasite
Source: PLoS Pathog. 2010 Oct 28;6(10):e1001165. doi: 10.1371/journal.ppat.1001165 (PMC2965767; doi:10.1371/journal.ppat.1001165)
Supplement: Figure S6 — Correlation of ApiAP2 mRNA abundance and expression of putative target genes. Average targets represents the average mRNA abundance profiles during the IDC for all genes in Dataset S5. mRNA abundance profile data was taken from [7]. The correlation coefficients are provided in the bottom right of each plot. (1.01 MB PDF) [file ppat.1001165.s007.pdf]

Positive Correlation between ApiAP2 and putative target genes

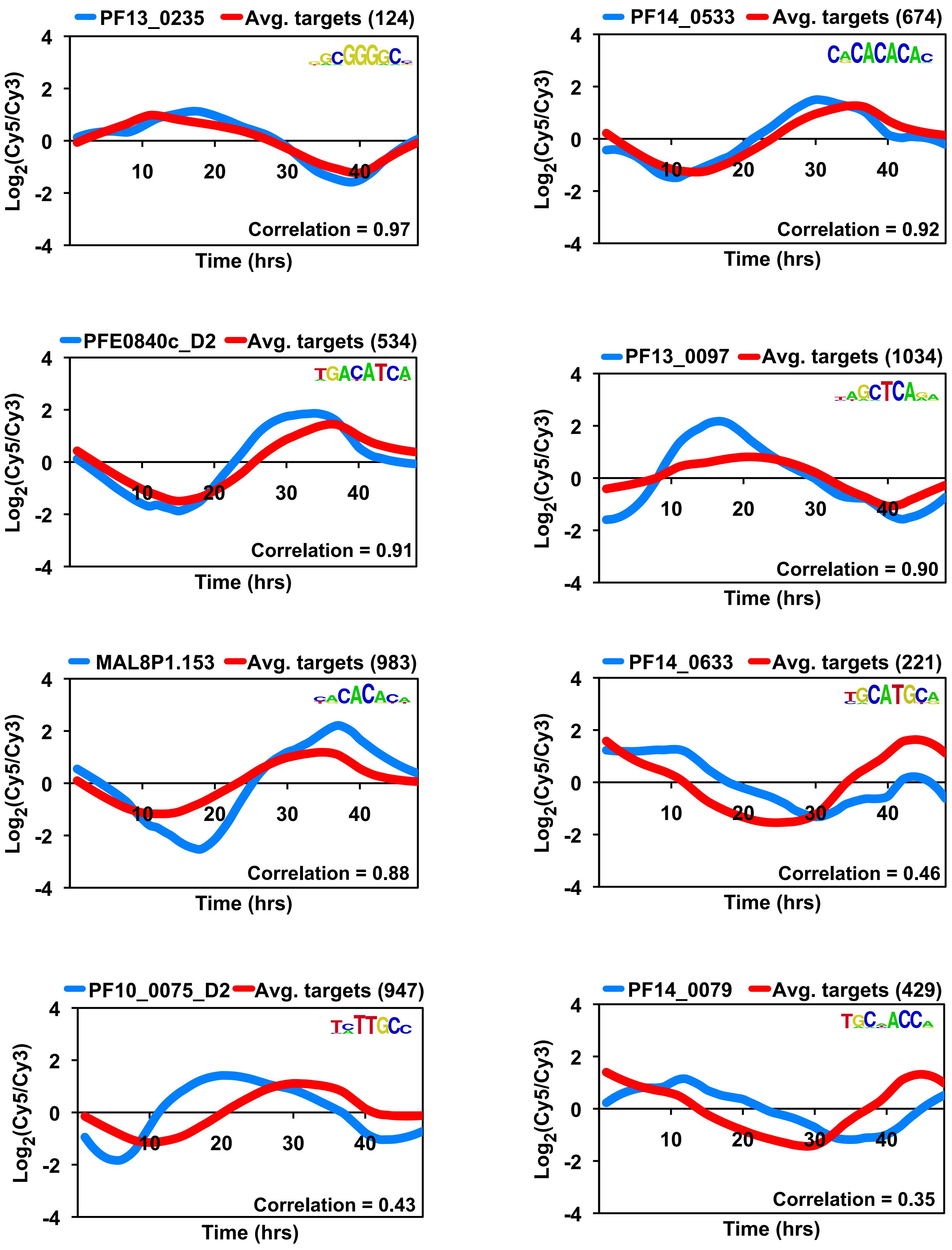

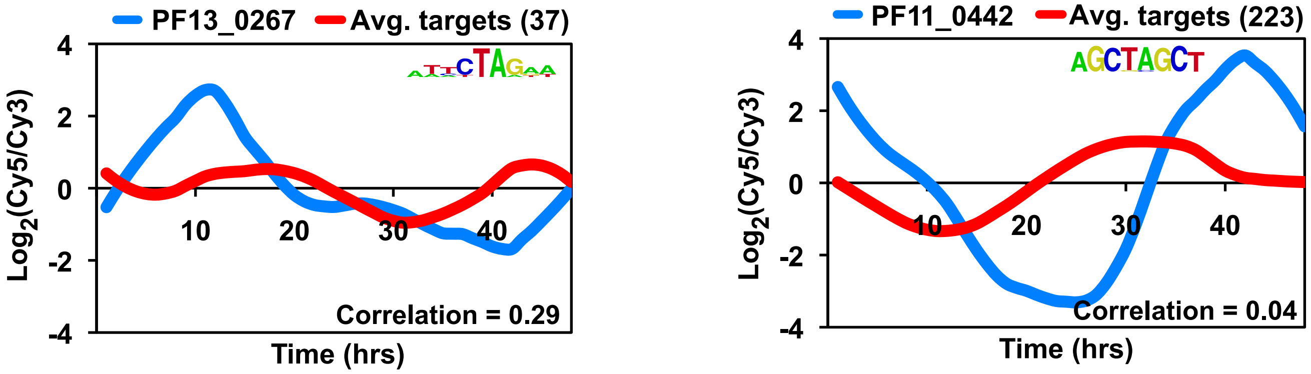

<u>Negative Correlation between ApiAP2 and putative target genes</u>

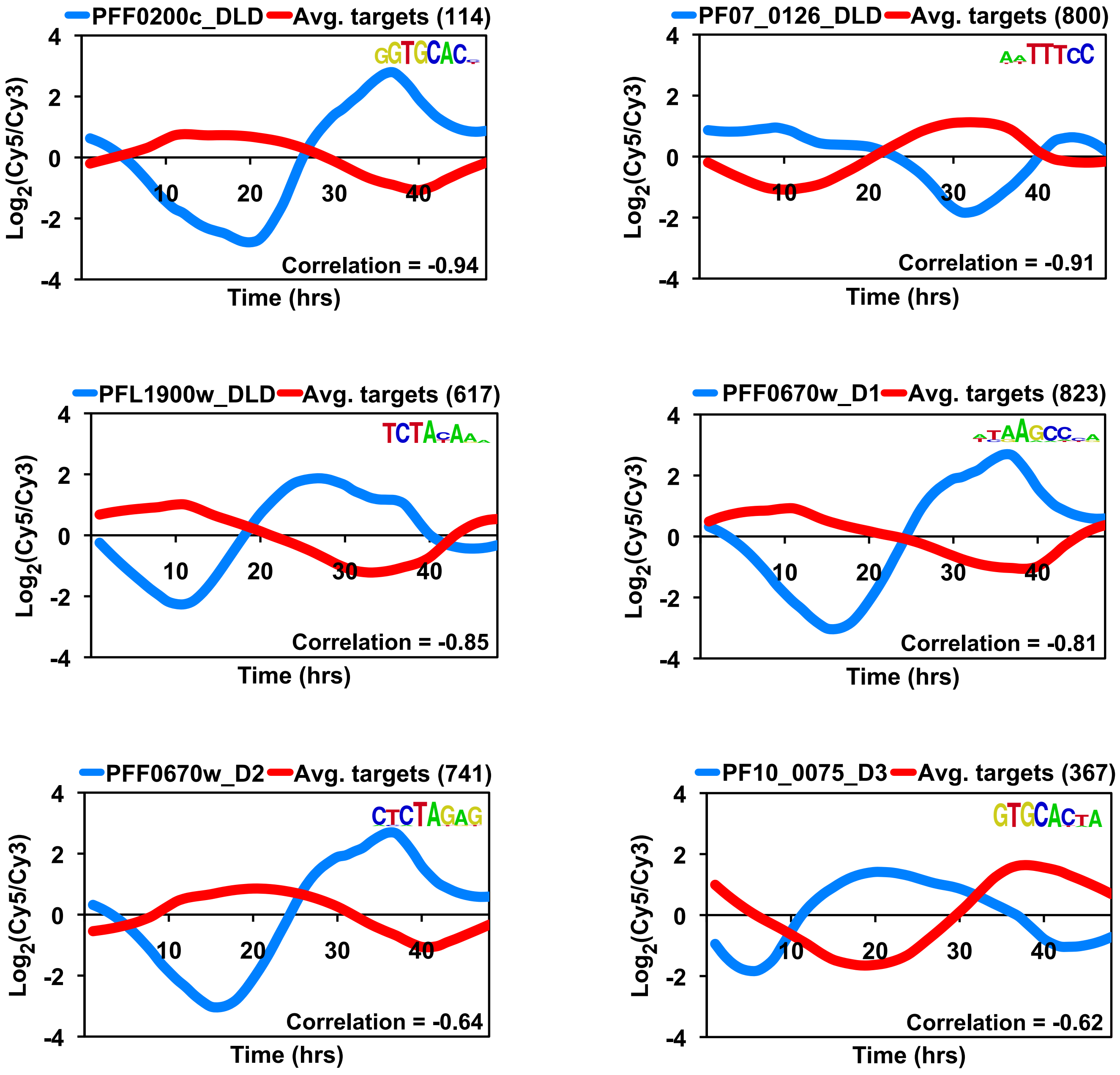

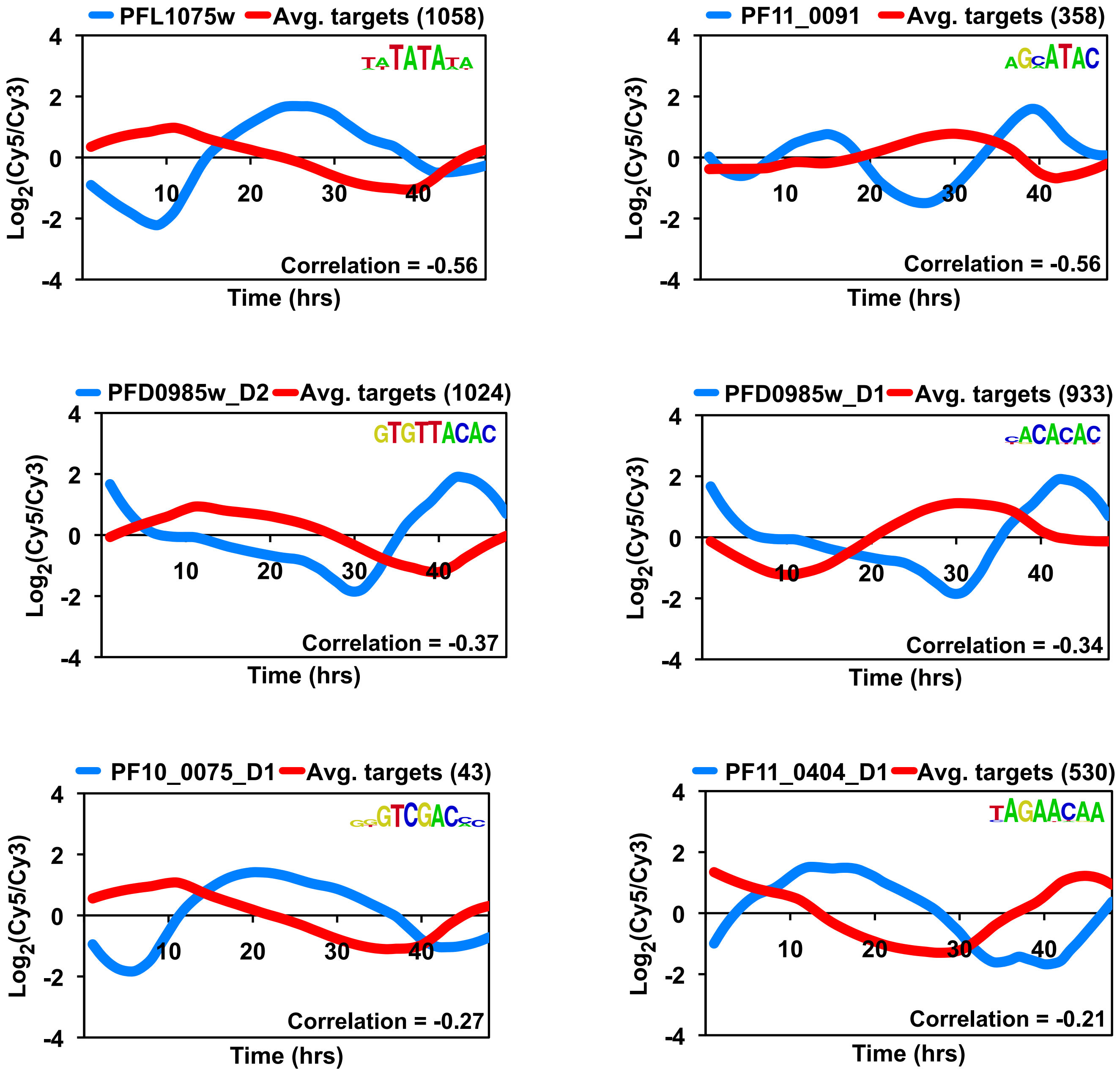
PFL1075w
Avg. targets (1058)
Correlation = -0.56
PF11_0091
Avg. targets (358)
Correlation = -0.56
PFD0985w_D2
Avg. targets (1024)
GTGTTACAC
Correlation = -0.37
PFD0985w_D1
Avg. targets (933)
Correlation = -0.34
PF10_0075_D1
Avg. targets (43)
Correlation = -0.27
PF11_0404_D1
Avg. targets (530)
Correlation = -0.21
Log2(Cy5/Cy3)
Time (hrs)
4
2
0
-2
-4
10
20
30
40
