# Supplementary material for: Identification and Genome-Wide Prediction of DNA Binding Specificities for the ApiAP2 Family of Regulators from the Malaria Parasite
Source: PLoS Pathog. 2010 Oct 28;6(10):e1001165. doi: 10.1371/journal.ppat.1001165 (PMC2965767; doi:10.1371/journal.ppat.1001165)
Supplement: Table S2 — Distribution and accessibility of ApiAP2 binding sites. (0.05 MB PDF) [file ppat.1001165.s016.pdf]

Table S2. Distribution and accessibility of ApiAP2 binding sites.

AP2 domain	Motif	Average number of motifs in all upstream regions	Total number of motif occurrences in the genome	Total number of motif occurrences in 2kb upstream regions	Number of motif occurrences in short read-mappable regions	Percent of motifs nucleosome free in 1 or more IDC timepoints	Percent of motifs nucleosome free in all 7 timepoints
PF10_0075_D1	GTCGAC	0.0	2726	189	176	73.9	5.1
PF13_0235_D1	GCCCCG	0.1	3093	434	412	64.6	1.7
PFF0200c_DLD	GTGCAC	0.2	3783	647	618	67.3	3.1
PF14_0633	GCATGC	0.3	6858	1407	1296	78.7	6.1
PF11_0442	GCTAGC	0.4	15 676	1741	1672	71.4	4.4
PF10_0075_D3	GTGCAC	0.5	9628	2102	2005	78.8	5.8
PF14_0533	CACACA	1.0	16 280	4304	4166	78.8	4.9
PF13_0026	CACACA	1.1	17 972	4746			
PFF0670w_D2	CTCTAG	2.2	68 743	9576	8813	83.8	7.9
PF13_0097	AGCTCA	3.3	99 203	13 965	13 375	83.7	6.8
PF14_0079	GCAACC	3.8	122 655	16 286	15 272	73.8	4.3
PF11_0091	GCATAC	5.8	95 747	25 068	23 481	89.7	9.7
PFL1085w	GTGTAC	6.3	139 161	26 845			
PFE0840c_D2	GACATC	7.8	247 785	33 168	31 199	82.9	6.5
PF10_0075_D2	TCTTGC	9.3	237 369	39 950	37 672	85.2	7.7
PFD0985w_D2	TGTTAC	11.1	220 017	47 780	43 996	90.5	11.2
PFD0985w_D1	GTGTGT	12.9	233 796	56 164	52 965	85.5	7.7
PFF0670w_D1	TAAGCC	18.5	500 590	79 563	75 047	83.5	6.9
MAL8P1.153	ACACAC	20.6	404 831	89 304	83 926	84.3	7.2
PF11_0404_D1	AGAACA	21.8	415 395	93 819	88 675	90.4	10.4
PFL1900w_DLD	TCTACA	24.1	471 151	103 952	97 562	91.6	11
PF07_0126_DLD	ATTTCC	32.4	646 287	138 803	132 144	88.5	9.2
PF13_0267	CTAGAA	37.8	918 000	163 680	151 278	90	10.6
PFL1075w	TATATA	196.7	1 843 912	844 332	791 090	96.8	13.3
