# Supplementary material for: Identification and Genome-Wide Prediction of DNA Binding Specificities for the ApiAP2 Family of Regulators from the Malaria Parasite
Source: PLoS Pathog. 2010 Oct 28;6(10):e1001165. doi: 10.1371/journal.ppat.1001165 (PMC2965767; doi:10.1371/journal.ppat.1001165)
Supplement: Table S3 — Conservation of IDC ApiAP2 targets between P. falciparum and P. vivax. (0.06 MB PDF) [file ppat.1001165.s017.pdf]

Table S3. Conservation of IDC ApiAP2 targets between *P. falciparum* and *P. vivax*.

AP2 domain and motif	Number of targets in *P. falciparum* IDC	Number of targets with an orthologue in *P. vivax*	Number of orthologues with motif	Percent of targets conserved
PF10_0075_D1 GTCGAC	43	39	0	0
PF10_0075_D2 TCTTGC	949	890	1	0.1
PFE0840c_D2 GACATC	535	494	11	2.2
PFF0200c_DLD GTGCAC	114	99	3	3.0
PF13_0026 CACACA	245	214	7	3.3
PFL1075w TATATA	1058	935	49	5.2
PF14_0079 GCAACC	430	326	37	11.3
PFL1085w GTGTAC	926	865	112	12.9
PF13_0267 CTAGAA	37	28	5	17.9
PF11_0404_D1 AGAACA	531	422	80	19.0
PFL1900w_DLD TCTACA	617	505	106	21.0
PF07_0126_DLD ATTTCC	802	763	174	22.8
PFD0985w_D1 GTGTGT	935	881	211	24.0
PFD0985w_D2 TGTTAC	1024	913	225	24.6
MAL8P1.153 ACACAC	986	921	244	26.5
PF11_0442 GCTAGC	223	207	55	26.6
PF10_0075_D3 GTGCAC	367	321	99	30.8
PF14_0533 CACACA	676	630	220	34.9
PF11_0091 GCATAC	359	344	124	36.0
PFF0670w_D2 CTCTAG	741	662	274	41.4

PF13_0235_D1 GCCCCG	124	112	52	46.4
PF14_0633 GCATGC	221	171	83	48.5
PFF0670w_D1 TAAGCC	823	723	361	49.9
PF13_0097 AGCTCA	1034	947	498	52.6
