# Supplementary material for: Identification and Genome-Wide Prediction of DNA Binding Specificities for the ApiAP2 Family of Regulators from the Malaria Parasite
Source: PLoS Pathog. 2010 Oct 28;6(10):e1001165. doi: 10.1371/journal.ppat.1001165 (PMC2965767; doi:10.1371/journal.ppat.1001165)
Supplement: Table S4 — Oligonucleotides used in EMSAs. (0.08 MB PDF) [file ppat.1001165.s018.pdf]

Table S4. Oligonucleotides used in EMSAs.

Oligo name	Sequence*
PFD0985w_D2-1°	AATATTATAATAGTCGTAGCCATCAAT**GTGTAACAC**ATGGTAATATAGATTTTCGTTTATATT
PFD0985w_D2-2°	AATATTATAATAGTCGTAGCCATCAAT**CCACACACC**ATGGTAATATAGATTTTCGTTTATATT
No motif	AATATTATAATAGTCGTAGCCATCAAT**AAAAAAAA**ATGGTAATATAGATTTTCGTTTATATT
PFL1900w_DLD-1°	AATATTATAATAGTCGTAGCCATCAAT**TCTACAAA**ATGGTAATATAGATTTTCGTTTATATT
PFL1900w_DLD-2°	AATATTATAATAGTCGTAGCCATCAAT**ACGACATC**ATGGTAATATAGATTTTCGTTTATATT
PFL1900w_DLD-3°	AATATTATAATAGTCGTAGCCATCAAT**ACCGCGGT**ATGGTAATATAGATTTTCGTTTATATT
hsp86	TTTTAAAAAAAAAAAAAAA**GCCCCG**CGGAA**AGGGGC**CATTGGATATATATTTAGTATTCC
hsp86-MUT1	TTTTAAAAAAAAAAAAAAA**GTATAG**CGGAA**AGGGGC**CATTGGATATATATTTAGTATTCC
hsp86-MUT2	TTTTAAAAAAAAAAAAAAA**GCCCCG**CGGAA**ATATAC**CATTGGATATATATTTAGTATTCC
hsp86-MUT3	TTTTAAAAAAAAAAAAAAA**GTATAG**CGGAA**ATATAC**CATTGGATATATATTTAGTATTCC
hsp70	TTTATATTATATATATATAATAATTTAG**GCCCCG**T**GCCC**TTATTTTGATTTTTTTTTTTT
pfc0975c	TTATGATGATACATAAATTACTGATCTTT**CTAG**GTGCGCTGCTACCATTATCGTGAATAA
pfc0975c-MUT	TTATGATGATACATAAATTACTGATCTTT**CAAA**GTGCGCTGCTACCATTATCGTGAATAA
mal8p1.107	AATATTTTTCGACAAGGTGCAGAGCAGTTT**CTAG**AATCAACACAGCGTTTCTTCTATCATTTG
mal8p1.107-MUT	AATATTTTTCGACAAGGTGCAGAGCAGTTT**CTAA**AATCAACACAGCGTTTCTTCTATCATTTG
rhopH3	ATTAATTTAATATGTACGCAAG**GTGCAC**ACTG**GTGCA**TTAAAAAAAAAATATATATTAAT
rhopH3-MUT1	ATTAATTTAATATGTACGCAAG**GTAAAC**ACTG**GTGCA**TTAAAAAAAAAATATATATTAAT
rhopH3-MUT2	ATTAATTTAATATGTACGCAAG**GTGCAC**ACTG**GTAAA**TTAAAAAAAAAATATATATTAAT
msp1	AAATTGTGTAAATTCATATTCGATAAA**GTGCA**TTAATTTTATTTATCAATTGTAAACATA
msp1-MUT	AAATTGTGTAAATTCATATTCGATAAA**GTTAA**TTAATTTTATTTATCAATTGTAAACATA
msp10	TCAACAATTATTATATAATTAATTAGAA**GTGCA**TTAGGTAACAACTGTCGAATAAATATAT
msp10-MUT	TCAACAATTATTATATAATTAATTAGAA**GTTAA**TTAGGTAACAACTGTC

	GAATAAATATAT
gbp130	AAGCAAATAAGATATTCCAAAAAAAA**<u>GTATT</u>**CTAATGTGTACTAATAAACCTTTATATATAA

*Motif sequences are bolded and underlined.
